# Neuroendocrine Regulation of Stress‐Induced T Cell Dysfunction during Lung Cancer Immunosurveillance via the Kisspeptin/GPR54 Signaling Pathway

**DOI:** 10.1002/advs.202104132

**Published:** 2022-02-27

**Authors:** Su Zhang, Fangfei Yu, Anran Che, Binghe Tan, Chenshen Huang, Yuxue Chen, Xiaohong Liu, Qi Huang, Wenying Zhang, Chengbin Ma, Min Qian, Mingyao Liu, Juliang Qin, Bing Du

**Affiliations:** ^1^ Shanghai Frontiers Science Center of Genome Editing and Cell Therapy Shanghai Key Laboratory of Regulatory Biology Institute of Biomedical Sciences and School of Life Sciences Changning Maternity and Infant Health Hospital East China Normal University Shanghai 200241 China; ^2^ BRL Medicine Inc. Shanghai 201109 China; ^3^ Department of General Surgery Tongji Hospital School of Medicine Tongji University Shanghai 200065 China

**Keywords:** ERK5, GPR54, kisspeptin, stress, T cell exhaustion

## Abstract

Emerging evidence suggests that physiological distress is highly correlated with cancer incidence and mortality. However, the mechanisms underlying psychological challenges‐mediated tumor immune evasion are not systematically explored. Here, it is demonstrated that acute restraint (AR) increases the level of the plasma neuropeptide hormones, kisspeptin, and the expression levels of its receptor, *Gpr54*, in the hypothalamus, splenic and tumor‐infiltrating T cells, suggesting a correlation between the neuroendocrine system and tumor microenvironment. Accordingly, administration of kisspeptin‐10 significantly impairs T cell function, whereas knockout of *Gpr54* in T cells inhibits lung tumor progression by suppressing T cell dysfunction and exhaustion with or without AR. In addition, *Gpr54* defective OT‐1 T cells show superior antitumor activity against OVA peptide‐positive tumors. Mechanistically, ERK5‐mediated NR4A1 activation is found to be essential for kisspeptin/GPR54‐facilitated T cell dysfunction. Meanwhile, pharmacological inhibition of ERK5 signaling by XMD8‐92 significantly reduces the tumor growth by enhancing CD8^+^ T cell antitumor function. Furthermore, depletion of *GPR54* or *ERK5* by CRISPR/Cas9 in CAR T cells intensifies the antitumor responses to both PSMA^+^ and CD19^+^ tumor cells, while eliminating T cell exhaustion. Taken together, these results indicate that kisspeptin/GPR54 signaling plays a nonredundant role in the stress‐induced tumor immune evasion.

## Graphical abstract

The neuroendocrine system is demonstrated to play nonredundant roles in physiological distress induced tumor suppressive microenvironment and immune evasion through increasing kisspeptin/GPR54 signaling pathway. And inhibition or depletion of kisspeptin/GPR54 significantly reduced tumor formation by eliminating T cell exhaustion and intensified the antitumor immune responses implying a promising target for T cell mediated immune therapy.

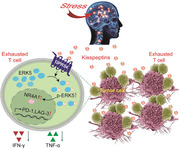

## 1 Introduction

Although the cancer death rate has fallen continuously from its peak in 1991 through 2018, with a total decline of 31%, cancer is still the second leading cause of death after heart disease in both men and women in the United States.^[^ ^1^ ^]^ The fast pace of life in the current society causes substantial anxiety and stress in people, leading to a variety of well‐known adaptive physiological changes, including changes in blood pressure, heart rate, endocrine output, neuronal activity, and the immune responses. Furthermore, chronic psychological stress is associated with poor prognosis in patients with cancer,^[^ ^2^ ^]^ and has also been proven to facilitate tumor progression and immune escape in various animal models. Chronic psychological stress also promotes lung carcinogenesis progression in mice,^[^ ^3^ ^]^ and stress hormones‐activated beta2‐adrenergic receptors accelerate resistance to the epidermal growth factor receptor inhibitors in nonsmall cell lung cancer.^[^ ^4^ ^]^ However, the underlying mechanism of stress‐induced immunosuppression in cancer development requires further exploration.

Chronic psychological stress leads to the activation of the hypothalamic‐pituitary‐adrenal (HPA) axis and sympathetic nervous system (SNS), along with the release of neurotransmitters or hormones, such as catecholamines and glucocorticoids.^[^ ^5^ ^]^ These neurotransmitters or hormones modulate the function of natural killer (NK) cells, cytotoxic T‐lymphocytes, dendritic cells (DCs), and macrophages in a paracrine manner.^[^ ^5^, ^6^ ^]^ T cells are the main effector cells that play critical roles in tumor progression, and previous studies have mainly focused on investigating the Th1/Th2 balance,^[^ ^7^ ^]^ dendritic cell maturation,^[^ ^8^ ^]^ and myeloid‐derived suppressor cell (MDSC) recruitment processes.^[^ ^9^ ^]^

When T cells are persistently exposed to tumor antigens in cancers, they are inclined to show exhausting phenotypes. Exhausted T cells lose robust antitumor functions and proliferative potential, express high and sustained multiple inhibitory receptors, and demonstrate metabolic dysregulation, poor memory recall and homeostatic self‐renewal, and distinct transcriptional and epigenetic programs.^[^ ^10^ ^]^ Inhibitory receptors (programmed cell death protein‐1 (PD‐1), T‐cell immunoglobulin and mucin domain‐3 (TIM‐3), lymphocyte activation gene‐3 (LAG‐3), cytotoxic T lymphocyte‐associated antigen‐4 (CTLA‐4), and T cell immunoglobulin and ITIM domain (TIGIT)) expressed on T cells can be induced and maintained by persistent T‐cell receptor (TCR) stimulation by factors, including hypoxia, cytokines (interferon (IFN)‐*γ*, interleukin (IL)‐2, IL‐7, IL‐15, and IL‐21), and transcription factors (T‐cell factor 1, eomesodermin, nuclear factor of activated T‐cells (NFAT), thymocyte selection‐associated high mobility group box (TOX), and nuclear receptor subfamily 4 group A (NR4A)).^[^ ^11^ ^]^ Although immunotherapies, such as immune checkpoint blockade therapy, adoptive cellular therapy, and cancer vaccinology, have become mainstay therapies for cancer treatment in recent years, their efficiency rates in solid tumors, especially low immunogenicity tumors, such as lung cancer, remain unsatisfactory.^[^ ^12^ ^]^ Only one‐third of patients with most types of cancer respond to immune checkpoint inhibitors. Adoptive T cell therapy has shown considerable promise in hematologic malignancies, but in solid tumors, it is less favorable due to immunosuppression, antigen escape, and physical barriers to the entry into solid tumors. Thus, understanding the mechanisms of T cell dysfunction and exhaustion is essential for establishing rational immunotherapeutic interventions.

G protein‐coupled receptor 54 (GPR54), also known as the KISS1 receptor (KISS1R), the key receptor for the neuropeptide hormone kisspeptin, plays an indispensable role in regulating puberty development and cancer metastasis.^[^ ^13^ ^]^ The activation of GPR54 by kisspeptin results in the activation of G*α*q/intracellular calcium ions (Ca^2+^), mitogen‐activated protein kinase, and phosphatidylinositol 3‐kinase/AKT pathways in a tissue‐specific manner.^[^ ^14^ ^]^ Our previous study indicated that kisspeptin/GPR54/calcineurin signaling in macrophages regulates a negative feedback loop of TBK1 signaling in the antiviral innate immune response.^[^ ^15^ ^]^ However, relatively little is known about the involvement of GPR54 in the antitumor immune response. Here, we demonstrated that kisspeptin/GPR54 signaling was significantly upregulated by psychological stress, which negatively regulated cancer immunosurveillance by promoting CD8^+^ T cell exhaustion. Most importantly, *Gpr54* knockout or the inhibition of downstream ERK5 signaling strengthens the antitumor responses of CD8^+^ T cells. Our results provide insight into the negative regulation of the antitumor immune response by the neuroendocrine system via the kisspeptin/GPR54/ERK5 axis‐mediated T cell dysfunction.

## 2 Results

### 2.1 Chronic Stress Negatively Affects Lung Cancer Immunosurveillance via Kisspeptin/GPR54

**Figure 1:**
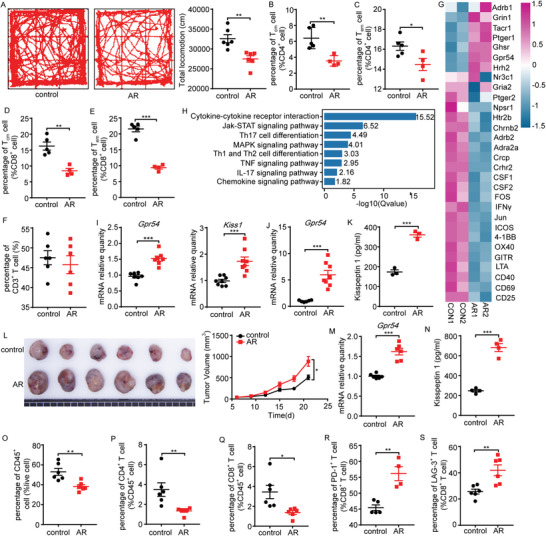
Chronic stress negatively affects lung cancer immunosurveillance via the kisspeptin‐KISS 1 receptor (KISS1R/GPR54) pathway. A) Representative locomotion tracks of the control and acute restraint (AR) mice in the open‐field test (OFT) and total locomotion (length of the track) were compared between the control and AR groups (*n* = 6). Flow cytometric (FCM) analysis of the proportions of B) splenic CD4^+^ central memory T cell (T_cm_), C) CD4^+^ effector memory T cell (T_em_), D) CD8^+^ T_cm_, E) CD8^+^ T_em_, F) CD3^+^ T (*n* = 4–6) cell populations on day 18 after AR treatment. C57BL/6 mice after 18 days of AR treatment, purified splenic CD3^+^ T cells activated by *α*‐CD3/*α*‐CD28 for 72 h, then harvested cells for RNA‐seq analysis. Heatmap shows G) the expression levels of neuropeptides or neurotransmitters and T cell activation related genes, and H) Kyoto Encyclopedia of Genes and Genomes (KEGG) enrichment analysis of the downregulated genes from the AR group compared with the control. Heatmap and KEGG analysis results were plotted using the OmicShare tools, a free online platform for data analysis (www.omicshare.com/tools). Gene expression levels of the hypothalamus *Gpr54* and *Kiss1* I) (*n* = 7–8) and J) splenic T cells *Gpr54* on day 18 after AR were determined by quantitative polymerase chain reaction (qPCR) (*n* = 8). K) Serum kisspeptin 1 levels after 18 days of AR were detected by enzyme‐linked immunosorbent assay (ELISA) (*n* = 3). C57BL/6 mice on day 18 after AR were then implanted with 10^6^ LLC tumor cells by a subcutaneous injection, and the mice were subjected to AR treatment daily. L) The LLC tumor growth curves and the end‐point tumor sizes were represented (*n* = 6), and M) *Gpr54* gene expression levels in the tumor‐infiltrating lymphocytes (TILs) were determined by qPCR (*n* = 6). N) Serum kisspeptin 1 levels at the tumor end‐point were detected by ELISA (*n* = 4). FCM analysis of the proportions of O) tumor CD45^+^, P) CD4^+^, Q) CD8^+^, R) PD‐1^+^ CD8^+^, and S) LAG‐3^+^ CD8^+^ cell populations at the tumor end‐point (*n* = 4–6). All data are from at least three independent experiments. Data are represented as the mean ± standard error of the mean (SEM). *P < 0.05, **P < 0.01, ***P < 0.001 by an unpaired Student's *t*‐test.

To investigate the potential impact of stress on cancer development and the tumor microenvironment (TME), we adopted an accepted acute restraint (AR) model as described.^[^ ^16^ ^]^ After 18 days of treatment, depression‐like behaviors were examined using the open‐field test (**Figure** **1**A), and the mice in the AR group exhibited significantly reduced total locomotion. After AR treatment, splenic central memory T cells (T_cm_) and effector memory T cells (T_em_) in CD4^+^ and CD8^+^ T cells (Figure 1B–E; Panel A in Figure S1, Supporting Information) were significantly reduced, accompanied by the expansion of naïve CD8^+^ T cells (Panel A in Figure S1, Supporting Information), Gr‐1^+^ MDSCs (Figure S2A, Supporting Information), and NK cells (Figure S2B, Supporting Information), while the ratio of CD3^+^ T cells (Figure 1F), DCs (Figure S2C, Supporting Information), and macrophages (Figure S2D, Supporting Information) were comparable (gating strategies in Figure S3, Supporting Information). The reason for the increase in NK cells may be that the NK cells were insensitive to stress‐induced apoptosis as iNKT cells.^[^ ^1^ ^]^

To investigate the influence of T cells in chronic restraint stress, splenic CD3^+^ T cells were analyzed by RNA sequencing (RNA‐seq). Chronic psychological stress has been reported to provoke the release of neurotransmitters and hormones, such as catecholamines and glucocorticoids.^[^ ^5^ ^]^ To explore the mechanistic link between AR and immunosuppression, we investigated the impact of AR on neuroendocrine stress mediators, such as corticosterone, serotonin, and norepinephrine. The heatmap showed the expression of neuropeptide/neurotransmitter receptor or T cell function/activated related genes (Figure 1G), and it was found that AR treatment upregulated *Gpr54* expression in T cells, whereas T cell activation‐related genes were significantly downregulated. RNA‐seq analysis revealed that the processes of cytokine–cytokine receptor interaction, JAK‐STAT signaling pathway, Th cell differentiation, and mitogen activated protein kinase (MAPK) signaling pathway were predominantly involved in AR‐induced immune suppression (Figure 1H). Interestingly, both *Kiss1* and *Gpr54* accumulated in the hypothalamus of AR mice (Figure 1I) as well as increased expression of *Gpr54* in splenic T cells (Figure 1J). In addition, serum levels of kisspeptin 1 (Figure 1K) were also elevated in AR mice, suggesting the neuroendocrine regulation of immunosurveillance by physiological distress.

Meanwhile, we also found increased tumor growth (Figure 1L), enhanced *Gpr54* expression in tumor‐infiltrating lymphocytes (Figure 1M), and higher serum levels of kisspeptin 1 (Figure 1N) in AR‐treated xenograft mice. Subsequently, we analyzed the changes in the immune cells in the tumor immune microenvironment. We discovered that the proportions of CD45^+^ cells (Figure 1O), CD4^+^ T cells (Figure 1P), and CD8^+^ T cells (Figure 1Q) all declined significantly after AR treatment, whereas the ratios of MDSCs (Figure S2E, Supporting Information), DCs (Figure S2F, Supporting Information), and NK cells (Figure S2G, Supporting Information) remained unchanged, and the proportion of macrophages was increased (Figure S2H, Supporting Information) (gating strategies in Figure S4 and Figure S5, Supporting Information).

To examine the role of the adaptive immune system in stress increased tumor development, we exposed recombination‐activating gene 2 (Rag2)^−/−^ mice to AR. The tumors in immunodeficient Rag2^−/−^ mice were almost uninfluenced by AR (Figure S2I, Supporting Information), implying that adaptive immunity is required for stress‐facilitated tumor growth. Additionally, CD8^+^ T cells in the tumor of the AR group showed increased expression of exhausted genes (PD‐1, LAG‐3) (Figure 1R,S). These data imply that kisspeptin/GPR54 signaling plays a dominant role in regulating CD8^+^ T cell dysfunction, which is fundamental in stress‐facilitated tumor growth.

### 2.2 GPR54 as a Poor Prognostic Factor for Lung Cancer

**Figure 2:**
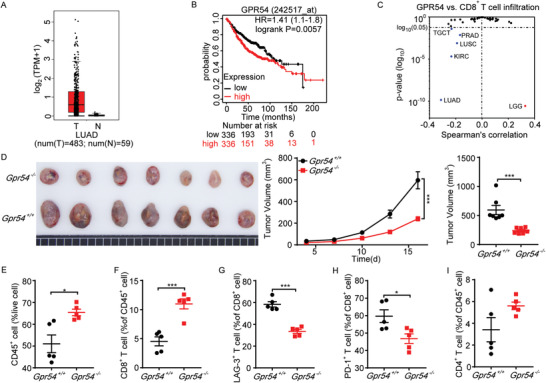
GPR54 promotes LLC tumor development and increases CD8^+^ T cell exhaustion. A) *GPR54* mRNA expression level data was extracted from The Cancer Genome Atlas (TCGA) lung adenocarcinoma (LUAD) dataset using Gene Expression Profiling Interactive Analysis (GEPIA) Cancer Genomics Browser. *GPR54* expression levels between human LUAD tissues (*n* = 483) and adjacent normal tissues (*n* = 59) was compared. B) Kaplan–Meier survival analysis showed that high *GPR54* (*KISS1R*) expression levels significantly correlated with poor overall survival of patients with LUAD. C) Scatter plots were generated using the Tumor Immune Estimation Resource (TIMER2.0) web tool of EPIC algorithm to identity CD8^+^ T cell infiltration that was associated with *GPR54* (*KISS1R*) expression levels in LUAD of TCGA database. D) *Gpr54* ^+/+^ and *Gpr54* ^−/−^ mice were implanted with 10^6^ LLC tumor cells by a subcutaneous injection. The LLC tumor growth curves and the end‐point tumor sizes were represented (*n* = 7). FCM analysis of the proportions of E) tumor CD45^+^, F) CD8^+^, G) LAG‐3^+^ CD8^+^, H) PD‐1^+^ CD8^+^, and I) CD4^+^ cell populations at the tumor end‐point (*n* = 5). All data are from at least three independent experiments. Data are represented as the mean ± SEM. *P < 0.05, **P < 0.01, ***P < 0.001 by an unpaired Student's *t*‐test or log(rank) test (B).

The Cancer Genome Atlas (TCGA) showed enhanced expression of *GPR54* in human lung cancer tissues (**Figure** **2**A). Meanwhile, higher *GPR54* expression in lung adenocarcinoma patients was significantly correlated with poor prognosis (Figure 2B), providing complementary support for the role of GPR54 in cancer development. Furthermore, *GPR54* expression was negatively associated with CD8^+^ T‐cell infiltration in lung adenocarcinoma (LUAD), kidney renal clear cell carcinoma, lung squamous cell carcinoma, and testicular germ cell tumors (Figure 2C). Only the expression of *GPR54* was associated with CD8^+^ T cell infiltration in lung cancer, although *Grin1* (glutamate receptor), *Ptger1* (prostaglandin E receptor 1), *Hrh2* (histamine receptor), *Ghsr* (growth hormone secretagogue receptor), and *Tacr1* (substance P receptor) were detected (Panel B in Figure S1, Supporting Information). These data demonstrate that GPR54 expression is associated with poor clinical outcomes in lung cancer.

To examine the effects of GPR54 on lung cancer development, we subcutaneously implanted LLC cells in *Gpr54* ^+/+^ (wild‐type) and *Gpr54* ^−/−^ mice (*Gpr54* knockout). Notably, *Gpr54* deficiency significantly restricted the subcutaneous tumor growth (Figure 2D). In addition, we observed a significant influence on the intratumoral immune cell composition in *Gpr54* ^−/−^ mice. The proportions of CD45^+^ cells (Figure 2E), CD8^+^ T cells (Figure 2F), and NK cells (Figure S6A, Supporting Information) were significantly increased in *Gpr54* ^−/−^ mice. Moreover, accompanied by fewer exhausted CD8^+^ T cells (Figure 2G,H) and MDSCs (Figure S6B, Supporting Information) infiltration, whereas CD4^+^ T cells (Figure 2I), DCs (Figure S6C, Supporting Information), and macrophages (Figure S6D, Supporting Information) showed little change. These data suggest that GPR54 promotes lung cancer development by suppressing the function of CD8^+^ T cells.

### 2.3 Kisspeptin‐10 Restricts the Function of CD8^+^ T Cell In Vitro

**Figure 3:**
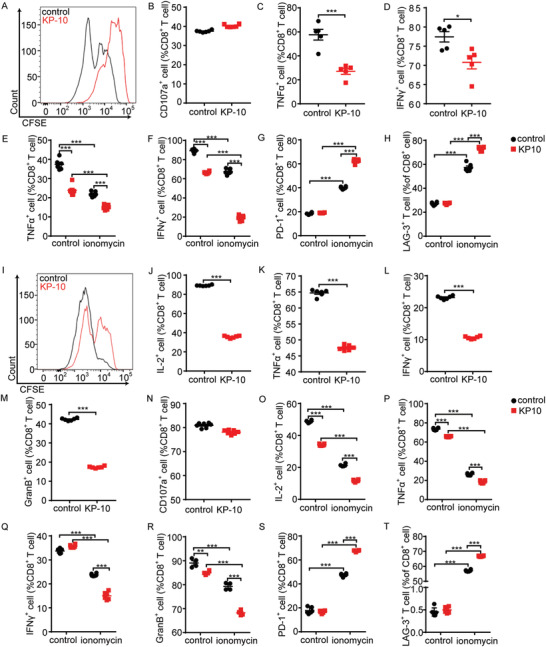
Kisspeptin impairs CD8^+^ T cell function. Mouse splenic CD8^+^ T cells were pretreated with KP‐10 (10 × 10^−6^ m) for 6 h, A) stained with carboxyfluorescein diacetate succinimidyl ester (CFSE), and the cell proliferation was measured 72 h later by FCM. B) CD107a with phorbol 12‐myristate 13‐acetate (PMA)/ionomycin plus protein transport inhibitor cocktail for 3 h then degranulation measured by FCM (*n* = 6). CD8^+^ T cells with PMA/ionomycin plus protein transport inhibitor cocktail for 4 h, flow cytometry measured C) TNF*α* and D) IFN*γ* release (*n* = 6). Mouse splenic CD8^+^ T cells were pretreated with KP‐10 (10 × 10^−6^ m) for 6 h, ionomycin for 16 h, then *α*‐CD3/*α*‐CD28 for 24 h, followed by E) TNF*α* and F) IFN*γ* release (*n* = 6) measured by FCM. Mouse splenic CD8^+^ T cells were pretreated with KP‐10 (10 × 10^−6^ m) for 6 h, ionomycin for 6 h, then FCM detection of G) PD‐1 and H) LAG‐3 expression levels (*n* = 6). Human peripheral CD8^+^ T cells were pretreated with KP‐10 (10 × 10^−6^ m) for 6 h, I) stained with CFSE, and cell proliferation were measured 72 h later by FCM. CD8^+^ T cells with PMA/ionomycin plus protein transport inhibitor cocktail for 4 h, J) FCM measured IL‐2, K) TNF*α*, L) IFN*γ*, and M) granzyme B (GranB) release (*n* = 6). N) CD107a with PMA/ionomycin plus protein transport inhibitor cocktail for 3 h then degranulation measured by FCM (*n* = 6). Human peripheral CD8^+^ T cells were pretreated with KP‐10 (10 × 10^−6^ m) for 6 h, ionomycin for 16 h, then PMA/ionomycin plus protein transport inhibitor cocktail for 4 h, followed by O) IL‐2, P) TNF*α*, Q) IFN*γ*, and R) GranB release (*n* = 6) measured by FCM. Human peripheral CD8^+^ T cells were pretreated with KP‐10 (10 × 10^−6^ m) for 6 h, ionomycin for 6 h, then S) FCM‐detected PD‐1 and T) LAG‐3 expression levels (*n* = 6). All data are from at least three independent experiments. Data are represented as the mean ± SEM. *P < 0.05, **P < 0.01, ***P < 0.001 by unpaired Student's *t*‐test (B–D, J–N) or one‐way analysis of variance (ANOVA) followed by least significant difference (LSD) analysis (E–H, O–T).

To further validate the role of the kisspeptin/GPR54 system in CD8^+^ T cells, we examined the functional alteration of CD8^+^ T cells by kisspeptin‐10 (KP‐10) in vitro. Compared with the control group, KP‐10 markedly inhibited the proliferation of CD8^+^ T cells (**Figure** **3**A) but slightly affected the degranulation of CD8^+^ T cells (Figure 3B). Moreover, KP‐10 significantly impaired TNF*α* (Figure 3C) and IFN*γ* (Figure 3D) release in CD8^+^ T cells (representative plots in Figure S7, Supporting Information). Accordingly, kisspeptin improved the expression of PD‐1 (Figure S6E, Supporting Information) and LAG‐3 (Figure S6F, Supporting Information) in *α*‐CD3 pretreated CD8^+^ T cells (representative plots in Figure S8E,F, Supporting Information). To further investigate the role of kisspeptin in CD8^+^ T cell exhaustion, we used an in vitro exhaustion system.^[^ ^18^ ^]^ The flow cytometry assay demonstrated that TNF*α* and IFN*γ* release were all decreased in KP‐10 treated cells (Figure 3E,F; representative plots in Figure S7, Supporting Information). On the contrary, the exhausted markers increased significantly (Figure 3G,H; representative plots in Figure S7, Supporting Information).

To confirm whether the presence of kisspeptin could influence the function of GPR54 in humans, CD8^+^ T cells from human peripheral blood mononuclear cells (PBMCs) were treated with KP‐10. Our results demonstrated that kisspeptin suppressed the proliferation of CD8^+^ T cells (Figure 3I) and the release of cytokines, including IL‐2 (Figure 3J), TNF*α* (Figure 3K), IFN*γ* (Figure 3L), and GranB (Figure 3M), but not their degranulation (Figure 3N) (representative plots in Figure S9, Supporting Information). When stimulated with ionomycin to induce T cell exhaustion, kisspeptin decreased cytokine release (Figure 3O–R), and strengthened the expression of exhausted markers (Figure 3S,T), suggesting that kisspeptin impaired CD8^+^ T cell function in vitro (representative plots in Figure S10, Supporting Information).

### 2.4 GPR54 Promotes CD8^+^ T Cell Exhaustion

**Figure 4:**
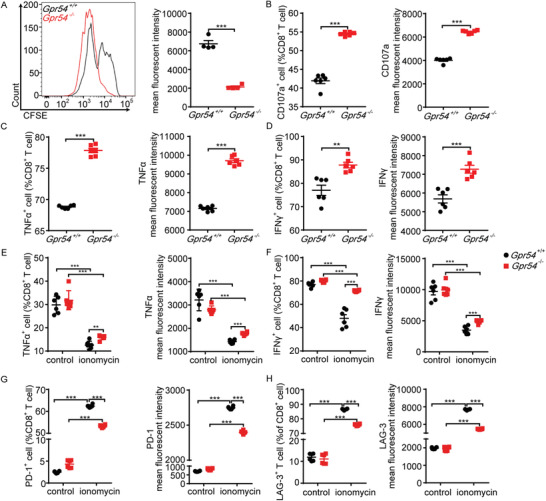
GPR54 promotes CD8^+^ T cell exhaustion in vitro. *Gpr54* ^+/+^ and *Gpr54* ^−/−^ mouse splenic CD8^+^ T cells activated with *α*‐CD3 (5 µg)/*α*‐CD28 (2 µg) for 72 h, proliferation A) measured by CFSE, B) degranulation detected with CD107a and PMA/ionomycin plus protein transport inhibitor for 3 h. CD8^+^ T cell with PMA/ionomycin plus protein transport inhibitor for 4 h, C) TNF*α* and D) IFN*γ* release detected by FCM. CD8^+^ T cell with ionomycin for 16 h, then *α*‐CD3/*α*‐CD28 for 24 h, E) TNF*α* and F) IFN*γ* release (*n* = 6) measured by FCM. CD8^+^ T cell with ionomycin for 16 h, then G) FCM detection PD‐1 and H) LAG‐3 expression levels (*n* = 6). All data are from at least three independent experiments. Data are represented as the mean ± SEM. *P < 0.05, **P < 0.01, ***P < 0.001 by unpaired Student's *t*‐test (A–D) or one‐way ANOVA followed by LSD analysis (E–H).

As described in Figure 2G,H, GPR54 has great potential to facilitate CD8^+^ T cell exhaustion in lung cancer. Therefore, we investigated the role of GPR54 in exhausted CD8^+^ T cells in vitro. As shown in **Figure** **4**, the proliferation (Figure 4A), degranulation (Figure 4B), and cytokine release (Figure 4C,D) all increased in *Gpr54* deficient CD8^+^ T cells (representative plots in Figure S11, Supporting Information). Additionally, *Gpr54* deprivation suppressed PD‐1 (Figure S6G, Supporting Information) and LAG‐3 (Figure S6H, Supporting Information) expression when CD8^+^ T cells were activated with *α*‐CD3 (representative plots in Figure S8G,H, Supporting Information). Accordingly, *Gpr54* knockout enhanced cytokine release (Figure 4E,F) and impaired the expression of inhibitory receptors (Figure 4G,H) in vitro (representative plots in Figure S11, Supporting Information). These results demonstrate that GPR54 suppresses CD8^+^ T cell function and enhances CD8^+^ T cell exhaustion.

**Figure 5:**
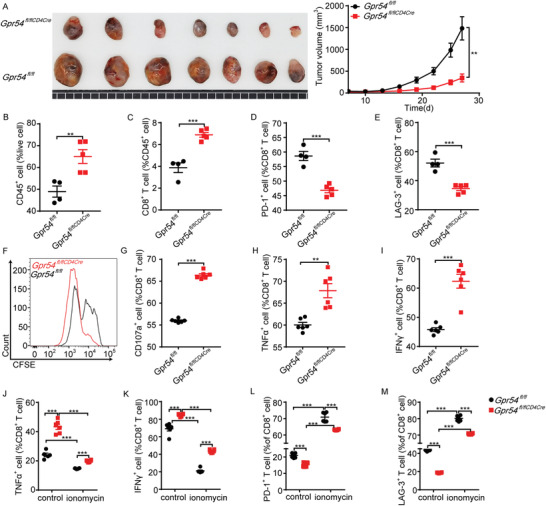
GPR54 T cell conditional knockout impairs LLC tumor development and decreases CD8^+^ T cell exhaustion. *Gpr54* ^fl/fl^ and *Gpr54* ^fl/flCD4Cre^ mice were implanted with 10^6^ LLC tumor cells by a subcutaneous injection. The LLC tumor growth curves and the end‐point tumor sizes were represented A) (*n* = 7). FCM analysis of the proportions of B) intratumoral CD45^+^, C) CD8^+^, D) PD‐1^+^ CD8^+^, and E) LAG‐3^+^ CD8^+^ cell populations at end‐point (*n* = 4–5). *Gpr54* ^fl/fl^ and *Gpr54* ^fl/flCD4Cre^ mouse splenic CD8^+^ T cells activated with *α*‐CD3 (5 µg)/*α*‐CD28 (2 µg) for 72 h, and F) proliferation measured by CFSE. G) Degranulation detected with CD107a and PMA/ionomycin plus protein transport inhibitor for 3 h. CD8^+^ T cell with PMA/ionomycin plus protein transport inhibitor for 4 h, H) TNF*α* and I) IFN*γ* release detected by FCM. CD8^+^ T cell treated with ionomycin for 16 h, then *α*‐CD3/*α*‐CD28 for 24 h, J) TNF*α* and K) IFN*γ* release (*n* = 6) measured by FCM. CD8^+^ T cell with ionomycin for 16 h, then L) FCM detected PD‐1 and M) LAG‐3 expression levels (*n* = 6). All data are from at least three independent experiments. Data are represented as the mean ± SEM. *P < 0.05, **P < 0.01, ***P < 0.001 by an unpaired Student's *t*‐ test (A–E, G–I) or one‐way ANOVA followed by LSD analysis (J–M).

To elucidate the influence of *Gpr54* deprivation on T cell function, we constructed *Gpr54* ^fl/flCD4Cre^ mice using the clustered regularly interspaced palindromic repeat/caspase 9 (CRISPR/Cas9) system. Consistent with conventional knockout data, specific deletion of *Gpr54* in T cells significantly impaired LLC tumor growth (**Figure** **5**A) and markedly increased intratumoral CD45^+^ cells (Figure 5B) and CD8^+^ T cells (Figure 5C). Likewise, reduced expression of PD‐1 (Figure 5D) and LAG‐3 (Figure 5E) was also observed (representative plots in Figure S12, Supporting Information). These data indicate that GPR54 deficiency reduces LLC tumor growth mainly by restricting CD8^+^ T cell exhaustion. Subsequently, we identified that *Gpr54* conditional deletion increased CD8^+^ T cell proliferation (Figure 5F), degranulation (Figure 5G), and cytokine release (Figure 5H,I) (representative plots in Figure S13, Supporting Information). *Gpr54* deletion also suppressed PD‐1 (Figure S6I, Supporting Information) and LAG‐3 (Figure S6J, Supporting Information) expression when CD8^+^ T cells were activated with α‐CD3 (representative plots in Figure S8I,J, Supporting Information). Enhanced cytokine release (Figure 5J,K) and decreased expression of inhibitory receptors (Figure 5L,M) were also found in the *Gpr54* knockout T cells in vitro exhaustion system (representative plots in Figure S13, Supporting Information). To further investigate the function of GPR54, we constructed an orthotopic lung tumor model. *Gpr54* conditional deletion in T cells impaired the growth of orthotopic lung tumors, decreased the weight of lung bearing tumors (Figure S14A,B, Supporting Information), and expression of inhibitory receptors in lung CD8^+^ T cells (Figure S14C,D, Supporting Information). These findings demonstrate that GPR54 increases LLC tumor growth by suppressing CD8^+^ T cell function and enhancing CD8^+^ T cell exhaustion.

### 2.5 Chronic Stress Promotes CD8^+^ T Cell Exhaustion via GPR54

**Figure 6:**
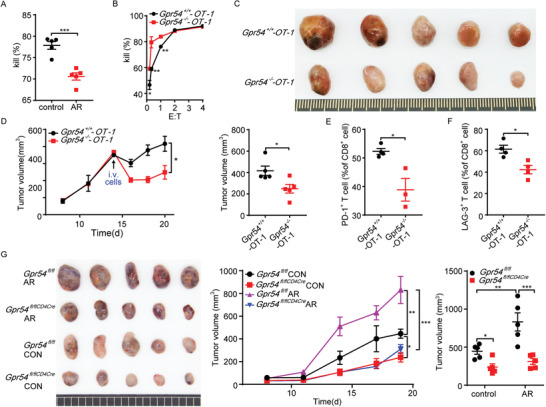
Chronic stress attenuates CD8^+^ T cell function via GPR54. OT‐1 mice with AR for 18 days, splenic CD8^+^ T cells activated with *α*‐CD3/*α*‐CD28, OT‐1 cells, and RM‐1‐ovalbumin (OVA)‐green fluorescent protein (GFP) cells cocultures with E:T = 1:2 for killing assessment A) (*n* = 5). *Gpr54* ^+/+^‐OT‐1 and *Gpr54* ^−/−^‐OT‐1 cells cocultured with RM‐1‐OVA‐GFP cells at indicated effector‐to‐target ratio for killing assessment B) (*n* = 6). Rag2^−/−^ mice were implanted with 10^6^ LLC‐OVA tumor cells by subcutaneous injection, followed by an intravenous injection of 10^6^ OT‐1 CD8^+^ T cells. Then, C) the end‐point tumor picture and D) tumor growth curves and tumor sizes were represented (*n* = 5). FCM analysis of the proportions of E) tumor PD‐1^+^ CD8^+^ and F) LAG‐3^+^ CD8^+^ cell populations after adoptively transferred OT‐1 cells (*n* = 4). G) *Gpr54* ^fl/fl^ and *Gpr54* ^fl/flCD4Cre^ mice on day 18 after AR implanted with 10^6^ LLC tumor cells by a subcutaneous injection, and the mice were subjected to AR treatment daily. LLC tumor growth curves and the end‐point tumor sizes are represented (*n* = 5). All data are from at least three independent experiments. Data are represented as the mean ± SEM. *P < 0.05, **P < 0.01, ***P < 0.001 by an unpaired Student's *t*‐test (A–F) or one‐way ANOVA followed by LSD analysis (G).

To explore whether GPR54 is involved in CD8^+^ T cell‐mediated cytotoxicity, OT‐1 CD8^+^ T cells were cocultured with RM‐1‐OVA‐GFP cells. This showed that chronic stress impaired T cell‐mediated cytotoxicity in tumor cells (**Figure** **6**A). Subsequently, OT‐1 mice subjected to AR and CD8^+^ T cells showed a better killing effect at low effector‐to‐target ratios after *Gpr54* deletion (Figure 6B). To further study the role of GPR54 in antigen‐specific CD8^+^ T cells, *Gpr54* ^+/+^‐OT‐1 and *Gpr54* ^−/−^‐OT‐1 cells were transferred into Rag2^−/−^ mice bearing LLC‐OVA tumors. Phenotypically, adoptively transferred *Gpr54* ^−/−^‐OT‐1 CD8^+^ T cells impaired LLC‐OVA tumor growth (Figure 6C,D), and decreased the expression of inhibitory receptors (Figure 6E,F) in CD8^+^ T cells (representative plots in Figure S15, Supporting Information). It is possible that chronic stress modulates CD8^+^ T cell function through GPR54 and subsequent tumor growth. To test this hypothesis, *Gpr54* ^fl/flCD4Cre^ mice were subjected to chronic stress and subcutaneous LLC tumors. We found that in normal state *Gpr54* conditional deletion impaired lung cancer growth, and chronic stress wild‐type mice LLC growth was significantly enhanced, but the tumor growth of *Gpr54* conditional deletion mice was mildly changed (Figure 6G). These results illustrate that GPR54 plays an important role in chronic stress‐induced CD8^+^ T cell exhaustion.

### 2.6 GPR54 Regulates CD8^+^ T Cell Exhaustion through the ERK5‐NR4A Signaling Pathway

**Figure 7:**
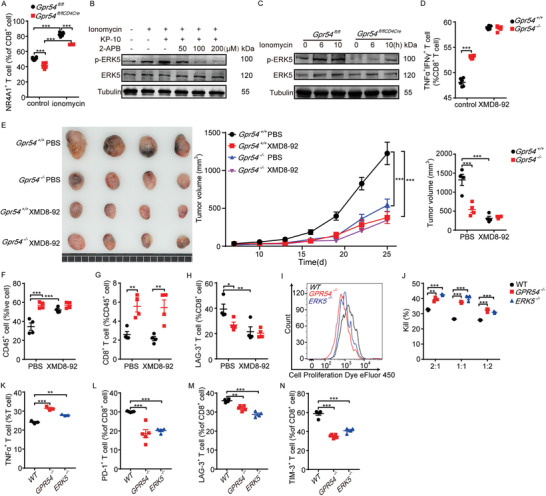
ERK5‐NR4A signaling pathway is involved in GPR54 regulated CD8^+^ T cell exhaustion. NR4A1 expression levels in the splenic CD8^+^ T cells with ionomycin (500 ng mL^−1^) were determined by FCM analysis A) (*n* = 6). B) CD8^+^ T cells were primed with ionomycin (500 ng mL^−1^) for 6 h, then pretreated with the indicated concentrations of 2‐APB for 1 h and stimulated with KP‐10 (50 × 10^−6^ m) for 1 h, and then ERK5 phosphorylation was analyzed by immunoblotting. C) ERK5 and p‐ERK5 expression levels in splenic CD8^+^ T cells with ionomycin (500 ng mL^−1^) were determined by Western blot. *Gpr54* ^+/+^ and *Gpr54* ^−/−^ mice splenic CD8^+^ T cells treated with XMD8‐92 (5 × 10^−6^ m) for 6 h, then with PMA/ionomycin plus protein transport inhibitor for 4 h, TNF*α* ^+^IFN*γ* ^+^ D) ratio detected by FCM (*n* = 6). E) *Gpr54* ^+/+^ and *Gpr54* ^−/−^ mice implanted with 10^6^ LLC tumor cells by a subcutaneous injection, the tumor volumes reached 50–70 mm^3^ and an intraperitoneal (i.p.) injection with XMD8‐92 (50 mg kg^−1^, twice a day) was given, the LLC tumor picture and tumor growth curves and tumor sizes with XMD8‐92 were represented (*n* = 4). FCM analysis of the proportions of F) tumor CD45^+^, G) CD8^+^, and H) LAG3^+^ CD8^+^ cell populations at end‐point (*n* = 4). I) PSMA‐CAR‐T cells were stained with 5 × 10^−6^ m of cell proliferation dye eFluor 450, and the cell proliferation was detected 72 h later by flow cytometry. PC3‐PSMA cells were mixed with the wild‐type (WT), *GPR54* ^−/−^, or *ERK5* ^−/−^ PSMA‐CAR‐T cells at the indicated effector‐to‐target (E:T) ratios, and J) cytotoxicity assay, K) cytokine release, and L–N) exhaustion are presented (*n* = 3–4). All data are from at least three independent experiments. Data are represented as the mean ± SEM. *P < 0.05, **P < 0.01, ***P < 0.001 by one‐way ANOVA followed by LSD analysis.

As nuclear orphan receptors are induced by various signals, the NR4A family has been demonstrated to be a key mediator of T cell dysfunction by decreasing the activity of AP‐1.^[^ ^19^ ^]^ We also observed that c‐fos and c‐jun, which are both involved in AP‐1 signaling, decreased noticeably in AR‐treated T cells. Therefore, we checked the expression of NR4A family and found a significant increase in *Nr4a1*, *Nr4a2*, and *Nr4a3* in exhausted CD8^+^ T cells, but this kind of enhancement was obviously reduced in *Gpr54* knockout T cells (Figure S16A, Supporting Information). Subsequently, flow cytometry revealed that *Gpr54* conditional deletion also impaired NR4A1 expression (**Figure** **7**A, representative plots in Figure S17, Supporting Information). Although previous data have shown that intracellular calcium is essential for G*α*q‐coupled G protein‐coupled receptors (GPCRs), as well as ERK5 phosphorylation, a direct correlation between ERK5 and GPR54 mediated immune function has been uncovered.^[^ ^15^, ^20^ ^]^ Thus, we analyzed ERK5 phosphorylation in ionomycin‐primed CD8^+^ T cells with or without kisspeptin or 2‐APB (to chelate intracellular Ca^2+^). We found that kisspeptin promoted ionomycin‐induced ERK5 phosphorylation, and this activation could be restricted by chelation of intracellular Ca^2+^ through 2‐APB (Figure 7B). Furthermore, *Gpr54* conditional deletion in T cells also decreased the ionomycin‐induced phosphorylation of ERK5 (Figure 7C). These data demonstrate that the ERK5‐NR4A1 pathway is involved in GPR54 mediated CD8^+^ T cell exhaustion. As the most recently identified member of the MAPK family, ERK5 displays several distinct structural and functional properties that set it apart from ERK1/2 and the other members of the MAPK family, which have been found to be crucial in activation of the NLRP3 inflammasome.^[^ ^20^ ^]^ To better evaluate the function of ERK5, CD8^+^ T cells were pretreated with the ERK5 inhibitor XMD8‐92. XMD8‐92 treatment enhanced cytokine release (Figure 7D, representative plots in Figure S17, Supporting Information), but there was no difference between *Gpr54* ^+/+^ and *Gpr54* ^−/−^ CD8^+^ T cells. Subsequently, to validate the function of ERK5 in vivo, we treated mice bearing LLC tumors with XMD8‐92. The results showed that XMD8‐92 treatment resulted in a distinct reduction in tumor burden, but the effect of XMD8‐92 disappeared when GPR54 was ablated (Figure 7E). Simultaneously, XMD8‐92 treatment increased the ratio of CD45^+^ cells (Figure 7F), but not CD8^+^ T cells (Figure 7G), and decreased CD8^+^ T cell exhaustion (Figure 7H) in *Gpr54* ^+/+^ mice. However, CD8^+^ T cell exhaustion showed little difference between *Gpr54* ^+/+^ and *Gpr54* ^−/−^ mice after XMD8‐92 treatment (representative plots in Figure S17, Supporting Information). These results imply that the ERK5 pathway is crucial for GPR54 modulated CD8^+^ T cell exhaustion.

To further examine the function of ERK5 and GPR54 in cancer immunotherapy, we generated *GPR54* or *ERK5* knockout PSMA‐CAR‐T cells (target of prostate cancer) using the CRISPR/Cas9 system. GPR54 and ERK5 deletion was detected by Western blot (Figure S18A,B, Supporting Information), and knockout of *GPR54* or *ERK5* enhanced PSMA‐CAR‐T cell proliferation (Figure 7I). Moreover, deletion of *GPR54* or *ERK5* in PSMA‐CAR‐T cells increased the cytotoxicity of PSMA‐overexpressing PC3 cells (human prostate adenocarcinoma cells) at a low effector‐to‐target ratio (Figure 7J), as well as TNF*α* release at an effector‐to‐target ratio of 1:1 (Figure 7K). Accordingly, PC3‐PSMA cell‐induced CAR‐T cell exhaustion was also decreased in PSMA‐CAR‐T cells (Figure 7L–N) (representative plots in Figure S19, Supporting Information). This is consistent with the above data, ERK5 depletion (Figure S18C, Supporting Information) in CD19‐CAR‐T cells did not influence CAR‐T cell proliferation (Figure S18D, Supporting Information), but enhanced cytotoxicity in Raji Burkitt lymphoma cells at low effector to target ratios (Figure S18E, Supporting Information), and increased cytokine release (Figure S18F,G, Supporting Information). Moreover, *ERK5* ^−/−^ CD19‐CAR‐T cells presented a lower exhausted phenotype in both tumor cells treated and KP‐10 induced deteriorated cells (Figure S18H,I, Supporting Information) (representative plots in Figure S20, Supporting Information). These results suggest that GPR54 or ERK5 depletion increases T cell function in CAR‐T cell therapy, which has great potential in adoptive cell therapy.

## 3 Discussion

Chronic stress is associated with aberrant persistent activation of SNS and the HPA axis, leading to enhanced production of cortisol and catecholamines.^[^ ^5^ ^]^ Previous studies have shown that SNSs play a crucial role in regulating immune cell development, such as myelopoiesis,^[^ ^21^ ^]^ and lymphocyte differentiation.^[^ ^22^ ^]^ Studies have shown that chronic stress promotes cancer progression by regulating DCs,^[^ ^3^ ^]^ NKs,^[^ ^5^ ^]^ iNKTs,^[^ ^17^ ^]^ MDSCs,^[^ ^23^ ^]^ and myeloid cells.^[^ ^24^ ^]^ However, the mechanism by which chronic stress affects CD8^+^ T cells in tumor immune microenvironments is not completely understood. Here, we found that chronic stress increases kisspeptin levels and activates GPR54 to promote lung cancer progression by enhancing the exhaustion of CD8^+^ T cells in a mouse model. Chronic stress leads to T cell exhaustion, high coinhibitory molecule expression, and low cytotoxic capacity. Therefore, our results provide novel insights into tumor T‐cell exhaustion and new therapeutic strategies for depression‐linked tumors.

Kisspeptin is a family of neuropeptides encoded by KISS1,^[^ ^13b^ ^]^ that mainly regulates puberty development and cancer metastasis. It has been documented that the expression of kisspeptin correlates with abnormal emotion, and a positive feedback mechanism may exist in the regulation of emotion.^[^ ^25^ ^]^ However, few studies have focused on the role of kisspeptin/GPR54 in modulating the cancer immune microenvironment with neuropsychiatric abnormalities. We demonstrated that chronic stress compromises the cytotoxicity of CD8^+^ T cells, and promotes tumor cell escape from immunosurveillance. Mechanistically, this hyperresponsive state is dependent on kisspeptin/GPR54 signaling in CD8^+^ T cells, which increases the exhaustion of CD8^+^ T cells. Thus, our data demonstrate a new immune mechanism in which kisspeptin/GPR54 facilitates tumor progression, GPR54 may act as a bridge between the neuroendocrine and immune systems.

Intracellular calcium interacts with calcineurin, leading to the dephosphorylation of NFAT and the formation of NFAT:AP‐1 complexes to activate T cells. By contrast, overwhelmingly enriched NFAT binding to the promoter of inhibitory receptors and transcription factors (TOX and NR4A) induced T cell exhaustion.^[^ ^26^ ^]^ Our earlier study reported that kisspeptin activating GPR54 induced calcineurin binding to the GPR54 cytoplasmic domain,^[^ ^15^ ^]^ but in T cells, the role of GPR54 was indistinct. To date, more than 800 GPCRs have been identified in humans that are only coupled with several types of G proteins. As a classic G*α*q‐coupled GPCR, GPR54 elevates levels of the ubiquitous second messenger, cytosolic Ca^2+^, in a PLC‐*β*‐dependent manner. Our previous data showed that P2Y1/G*α*q mediated ERK5 phosphorylation is essential for activation of the NLRP3 inflammasome.^[^ ^20^ ^]^ However, the function of ERK5 in T cell dysfunction and the correlation between TCR/NFAT and G*α*q/PLC‐*β* signaling in the regulation of T cell exhaustion is not well understood. Actually, the function of ERK5 is mainly found in sustaining the survival and proliferation of tumor cells to accelerates tumor growth. Thus, this will be exciting to confirm whether ERK5 could facilitate tumor development by restricting cancer immunosurveillance. Our findings demonstrated that ERK5 did not affect the proliferation of activated T cells, however, it remarkably enhanced T cell exhaustion, suggesting a new mechanism by which calcium regulates T cell exhaustion.

Despite the remarkable results of CAR‐T cell therapy in a small subset of patients with hematologic malignancies, dysfunction due to T cell exhaustion has become an important barrier to progress.^[^ ^27^ ^]^ Mechanistically, impaired CAR‐T cell efficiency includes limited T cell persistence, such as T cell exhaustion and activation‐induced cell death. Thus, avoiding overactivation‐induced CAR‐T cell exhaustion could improve the efficiency of CAR‐T cell therapy especially in solid tumors. To investigate the role of kisspeptin/GPR54 signaling in T‐cell‐mediated immune therapy, we knocked out *GPR54* or *ERK5* in conventional CD19‐CAR‐T cells or PSMA‐CAR‐T cells. Our findings suggest that GPR54 or ERK5 deficient CAR‐T cells exert a favorable killing effect at low effector‐to‐target ratios and lower CAR‐T cell exhaustion, highlighting the promising clinical potential of GPR54 or ERK5 inhibition in CAR‐T cell immunotherapy for human cancer.

In summary, we demonstrated the crucial role of kisspeptin/GPR54 signaling in chronic stress‐induced immune suppression in the tumor microenvironment. Depletion of kisspeptin/GPR54 signaling or downstream ERK5 signaling not only rescued the chronic stress‐induced tumor growth but also strengthened T cell‐mediated immune therapy by restricting T cell exhaustion. Thus, our data demonstrate the great potential of kisspeptin/GPR54 signaling as a target for improving T cell‐mediated immune therapy in clinical settings.

## 4 Conclusion

In this study, we found that AR treatment increased the levels of the plasma neuropeptide hormone, kisspeptin, and the expression levels of its receptor, *Gpr54*, in the hypothalamus, T cells, and tumor‐infiltrating T cells, suggesting a correlation between the neuroendocrine system and tumor microenvironment. Moreover, the kisspeptin/GPR54 pathway promoted lung tumor progression and enhanced T cell dysfunction and exhaustion, with or without AR. Mechanistically, ERK5‐mediated NR4A1 activation was found to be essential for kisspeptin/GPR54‐facilitated T cell dysfunction, suggesting a new role for GPCR‐mediated G*α*q signaling in T cell dysfunction.

## 5 Experimental Section

### Mice

Male C57BL/6 mice aged 6–8 weeks were purchased from Jihui Laboratory Animal Care Co., Ltd (Shanghai, China). OT‐1, CD4Cre, and Rag2^−/−^ mice were purchased from Jackson Laboratories (Bar Harbor, USA). *Gpr54* ^fl/fl^ mice were constructed using the CRISPR/Cas9 system. *Gpr54*‐deficient mice (C57BL/6) were generated as previously described.^[^ ^18^ ^]^ All mice were maintained in specific‐pathogen‐free conditions under a 12 h light/12 h dark cycle following governmental and institutional guidelines (Laboratory Animal Welfare and Ethics Committee of the East China Normal University) for animal welfare. All experiments were approved by the Laboratory Animal Welfare and Ethics Committee of the East China Normal University (m20210602 for animal experiments).

### Cell Lines and Cell Culture

LLC, RM‐1, and Raji cell lines were obtained from the Stem Cell Bank, Chinese Academy of Sciences (Shanghai, China). LLC cells were cultured in Dulbecco's modified Eagle medium (Gibco, 11965092) supplemented with 10% fetal bovine serum (FBS, Gibco, 10099141), 1% nonessential amino acid (Gibco, 11140050), and 1% penicillin/streptomycin (P/S, Gibco, 15070063). Raji and RM‐1 cells were cultured in the Roswell Park Memorial Institute 1640 (RPMI‐1640, Gibco, 22400089) medium supplemented with 10% FBS and 1% P/S. All cell lines were routinely tested to confirm the absence of *Mycoplasma* contamination using the MycAway Plus‐Color One‐Step Mycoplasma Detection Kit (Yeasen Bio‐technol) and the most recent test date for all cells was November 28, 2021. All cell lines were used within ten generations after thawing in all the experiments.

### Acute Restraint Stress Model

Mice were restricted to a 50 mL tube for 2 h per day (from 9:00 to 11:00) for 18–40 consecutive days. Control mice were maintained in their home cages. Blood samples were collected at the end of the experiment and stored at −80 °C before further analysis.

### Behavioral Tests

The open field was a square chamber (40 × 40 cm), which was placed in the center and monitored for 5 min with an overhead video tracking system (Chengdu Techman Software Co., Ltd) that recorded the animal's location and path and the time the animal spent in the center square.

### Tumor Models

Wild type, Rag2^−/−^, *Gpr54* ^−/−^, *Gpr54* ^fl/fl^, *Gpr54* ^fl/flCD4Cre^ 6–8 weeks male mice were implanted with 10^6^ tumor cells (LLC or LLC‐OVA) by a subcutaneous injection, and the tumor growth was monitored for up to 25 days. Tumor size was measured every 2–3 days after the tumors were palpable or after the indicated treatment. Tumor volumes were calculated using the equation (l × w^2^)/2. Wild type and *Gpr54* ^−/−^ mice with LLC tumors were treated with the ERK5 inhibitor, XMD8‐92 (50 mg kg^−1^, MCE, HY‐14443), or PBS by an intraperitoneal injection twice a day when tumor volumes reached 50–70 mm^3^ for 13 days. For the lung orthotopic tumor model, 2 × 10^5^ LLC cells were mixed at a 1:1 ratio with Matrigel (Corning, 356237) in a final volume of 50 µL, and injected into the left lung lobe by an intercostal injection at the median axillary line of *Gpr54* ^fl/fl^ and *Gpr54* ^fl/flCD4Cre^ mice. Lung bearing tumors were collected on day 10 after engraftment.

### Cell Isolation

Freshly recovered tumors and spleens were dissected, minced, digested with 10 U mL^−1^ collagenase I (Gibco, 17100017), 400 U mL^−1^ collagenase IV (Gibco, 17104019), and 30 U mL^−1^ DNase I (Sigma‐Aldrich, D5025) in serum‐free RPMI‐1640 medium. These tissues were incubated at 37 °C for 30 min (tumor), and the cell suspension was filtered with 70 µm cell strainers. Red blood cells were solubilized with the red cell lysis buffer, and the resulting suspension was filtered through a cell strainer to produce a single‐cell suspension. Cells were washed once with PBS before use in flow cytometry analysis or magnetic bead purification.

### CD8^+^ T Cell Isolation and Culture

Spleen CD8^+^ T cells were purified using the EasySep mouse CD8a positive selective kit (STEMCELL, 18953). CD8^+^ T cells were cultured with plate‐bound *α*‐CD3 (5 µg mL^−1^, PeproTech, 17A2), soluble *α*‐CD28 (2 µg mL^−1^, PeproTech, 37.51), and IL‐2 (30 U mL^−1^, PeproTech, 212‐12) for 72 h in a complete T cell media (X‐VIVO [Lonza, 04‐418Q] supplemented with 10% FBS and 1% penicillin/streptomycin).

### Western Blotting

Activated CD8^+^ T cells were rested for 1 day and serum starved for 12 h, then incubated with ionomycin (500 ng mL^−1^, MCE, HY‐13434) for 6 and 10 h. Cells were treated with the radioimmunoprecipitation assay buffer (Beyotime, P0013B), supplemented with a protease and phosphatase inhibitor cocktail (Cwbio, CW2200 and CW2383). Lysates from 0.5 million cells from each group were run on a 10% polyacrylamide gel. ERK5 antibody (Cell Signaling Technology, 3372, 1:1000), phospho‐ERK5 (Thr218/Tyr220) antibody (Cell Signaling Technology, 3371, 1:1000), KISS1R (Affinity Biosciences, DF7123, 1:500), and *β*‐Tubulin (Abmart, M30109, 1:50 000) were used for detection, and analysis was performed using ODYSSEY CLX (LI‐COR).

### RNA Isolation and qRT‐PCR Analysis

Total RNA was isolated using TRIzol reagent. RNA (500 ng) was used for cDNA synthesis (PrimeScript RT Master Mix, Takara, RR036A). qRT‐PCR was performed using a Light Cycler 480II Real‐Time PCR system (Roche). The following primers were used: *Gapdh* forward: 5′‐CCAGGCGGCACGTCAGATCC‐3′, *Gapdh* reverse: 5′‐AAGCTGTGGCGTGATGGCCG‐3′; *Nr4a1* forward: 5′‐CCGGTGACGTGCAACAATTT‐3′, *Nr4a1* reverse: 5′‐CGGGTTTAGATCGGTATGCCA‐3′; *Nr4a2* forward: 5′‐TCGGTTTACTACAAGCCCTCT‐3′, *Nr4a2* reverse: 5′‐GGGGCGACTGCTTAAAGGA‐3′; *Nr4a3* forward: 5′‐TATGGCTCGGAATACACCACA‐3′, *Nr4a3* reverse: 5′‐ GCCCTCCATGAAGGTACTGAA‐3′; *Gpr54* forward: 5′‐ TACATCGCTAACCTGGCTGC‐3′, *Gpr54* reverse: 5′‐CCCAGATGCTGAGGCTGAC‐3′; *Kiss1* forward: 5′‐ CGAAGGAGTTCCAGTTGTAGG‐3′, *Kiss1* reverse: 5′‐AAGGAATCGCGGTATGCA‐3′. All data were normalized to GAPDH expression, and relative gene expression was quantified using the 2^−ΔΔCt^ method.

### T Cell Proliferation Assay

Activated CD8^+^ T cells from *Gpr54* ^+/+^, *Gpr54* ^−/−^, *Gpr54* ^fl/fl^, and *Gpr54* ^fl/flCD4Cre^ mouse spleens were labeled with CFSE and washed twice with PBS. On day 3, the cells were analyzed by flow cytometry to quantify the ratio of proliferating cells. For KP‐10 analysis, CD8^+^ T cells were pretreated with 10 × 10^−6^ m KP‐10 (GL Biochem, Shanghai) for 6 h, then labeled with CFSE.

### T Cell Degranulation Assay

Activated CD8^+^ T cells were stimulated with PMA/ionomycin plus protein transport inhibitor cocktail by adding 2 µg mL^−1^ antimouse CD107a (LAMP‐1) (1D4B, BioLegend, 1:200) for 3 h. Cells were washed once with the staining buffer (PBS with 2% FBS) and then analyzed by flow cytometry. For KP‐10 analysis, CD8^+^ T cells were pretreated with 10 × 10^−6^ m of KP‐10 for 6 h and then labeled with the antimouse CD107a (LAMP‐1) (1D4B, BioLegend) or antihuman CD107a (H4A3, BioLegend, 1:200) antibody.

### Serum Kisspeptin 1 Analysis

All blood samples were collected in nonheparinized tubes, clotted at room temperature for 2 h, and then centrifuged at 3000 rpm for 30 min at 4 °C. Serum kisspeptin 1 was analyzed using a mouse kisspeptin 1 ELISA kit (MyBioSource, MBS2533487) according to the manufacturer's instructions.

### Preparation of CAR‐T Cells

Peripheral blood was obtained from healthy volunteers (*n* = 3 or more) and statements that informed written consent of all participants were obtained. All blood samples were collected and handled according to the ethical and safety procedures approved by the Clinical Ethics Committee of the First Affiliated Hospital, College of Medicine, Zhejiang University (IIT20210001C‐R1 for human subjects). Human CD4^+^ and CD8^+^ T cells were purified from PBMCs using human CD4 (Miltenyi, 130‐045‐101) and CD8a magnetic MicroBeads (Miltenyi, 130‐045‐201). T cells were cultured with human T cell TransAct (Miltenyi, 130‐111‐160) and IL‐2 (200 U mL^−1^, Huaxin Biotechnology) for 48 h in complete T cell media (X‐VIVO [Lonza] supplemented with 10% FBS and 1% penicillin/streptomycin). CAR constructs were generated by gene synthesis of single‐chain fragment variables (scFVs) specific to human CD19 and PSMA, a costimulatory domain 4‐1BB, and the CD3 zeta chain. Lentiviral transductions (MOI = 10) were performed in six‐well plates and spun at 1800 rpm for 2 h at 37 °C in a prewarmed centrifuge, and cells were infected in an incubator at 37 °C for 18 h. After transduction, *ERK5* ^−/−^ CD19 CAR‐T cells were constructed using the CRISPR/Cas9 system. First, 25 µg of Cas9 (Thermo, A36499) protein, 15 µg of sgRNA1, and 15 µg of sgRNA2 were incubated for 15 min at room temperature. Subsequently, the cells were washed with PBS, and 10^7^ cells were resuspended in 100 µL of P3 primary cell solution and quickly added to the Cas9 sgRNA RNP complex. The mixture was briefly mixed and then added to an electroporation chamber (Lonza, V4XP). Cells were electroporated with the program T cell stimulation using the Lonza Nucleofector. *ERK5* sgRNA uses two reported sequences (31): sgRNA1: 5′‐CTTCGATGTGACCTTTGACG(TGG)‐3′ and sgRNA2: 5′‐GCATGCGACCCAGCAGTGAT(AGG)‐3′. *GPR54* sgRNA sequences, sgRNA1: 5′‐AAGTTGGTCACGGTCCGCAT(CGG)‐3′, sgRNA2: 5′‐TGTGGCGCCAACGCCTCGGA(CGG)‐3′, sgRNA3: 5′‐GACTGGGCCGTCCGAGGCGT(TGG)‐3′.

### In Vitro Killing Assay

RM‐1‐OVA‐GFP cells (2 × 10^5^, target cells) were mixed with *Gpr54* ^+/+^‐OT‐1 or *Gpr54* ^−/−^‐OT‐1 CD8^+^ T cells at different ratios in 200 µL T cell media in each well of the 96‐well plates, with a lid flat bottom (Corning, 3474), and incubated for 10 h. The remaining RM‐1‐OVA‐GFP cells were analyzed using flow cytometry.

### Adoptive Transfer

LLC‐OVA cells (10^6^) were injected subcutaneously into 6–8 week old male Rag2^−/−^ mice. After 14 days, 10^6^ *Gpr54* ^+/+^‐OT‐1, and *Gpr54* ^−/−^‐OT‐1 cells were intravenously transferred into these mice. Tumor growth was monitored every other day. On day 20, intratumoral T cells were analyzed by flow cytometry.

### Induction of Ionomycin‐Induced T Cells Anergy In Vitro

A previously published protocol was adopted using ionomycin to induce anergy.^[^ ^18^ ^]^ Naïve CD8^+^ T cells were stimulated with *α*‐CD3 (5 µg mL^−1^, PeproTech) and *α*‐CD28 (2 µg mL^−1^, PeproTech) for 72 h. The cells were rested for 1 day and serum starved for 12 h, then incubated with ionomycin (500 ng mL^−1^, MCE) for 16 h. Cells were harvested for Super Bright 780‐PD‐1 (J43, 1:200), PerCP‐eFluor710‐LAG‐3 (C9B7W, 1:200), PE‐NR4A1 (12.14, 1:200) exhaustion detection, or washed twice with medium. Then, the cells were restimulated with *α*‐CD3 (1 µg mL^−1^, PeproTech) and *α*‐CD28 (1 µg mL^−1^, PeproTech) for 24 h, and eBioscience protein transport inhibitor cocktail (eBioscience, 00‐4980‐03) was added 4 h before harvesting the cells. The restimulated cells were stained with the FITC‐conjugated antimouse TNF*α* (MP6‐XT22, BioLegend, 1:200), APC‐conjugated antihuman/mouse GranB (QA16A02, BioLegend, 1:200), and PE‐conjugated antimouse IFN*γ* (XMG1.2, BioLegend, 1:200) antibodies.

### Flow Cytometry

Surface staining was performed with 2 µg per test of the following fluorochrome‐conjugated antibodies at 4 °C for 45 min. FITC‐conjugated antimouse CD45 (30‐F11, 1:200), APC‐conjugated antimouse CD8 (53‐6.7, 1:200), APC‐conjugated antimouse Gr‐1 (RB6‐8C5, 1:200), PE‐Cyanine7‐conjugated antimouse CD62L (MEL‐14, 1:200), PerCP/Cyanine5.5‐conjugated antimouse CD11c (N418, 1:200) antibodies were from BioLegend; eFluor506‐conjugated antimouse CD4 (RM4‐5, 1:200), Super Bright 600‐conjugated antimouse CD3 (145‐2C11, 1:200), Super Bright 645‐conjugated antimouse CD11b (M1170, 1:200), PE/Cy7‐conjugated antimouse F4/80 (BM8, 1:200), Super Bright 780‐conjugated antimouse I‐A/I‐E (M5/114.15.2, 1:200), Super Bright 645‐conjugated antimouse CD44 (1M7, 1:200), Super Bright 780‐conjugated antimouse PD‐1 (J43, 1:200), PerCP‐eFluor710‐conjugated antimouse LAG‐3 (C9B7W, 1:200), PE‐conjugated antimouse NR4A1 (12.14, 1:200), Alexa Fluor 700‐conjugated antimouse CD8 (53‐6.7, 1:200), APC‐eFluor780‐conjugated antihuman CD8 (RPA‐T8, 1:200), FITC‐conjugated antihuman LAG‐3 (3DS223H, 1:200), and PE‐conjugated antihuman PD‐1 (MIH4, 1:200) antibodies, and eBioscience Fixable Viability Dye eFluor 780 (65‐0865‐14, 1:300) were from Thermo Fisher Scientific; Alexa Fluor 700‐conjugated antimouse NK (PK/36, 1:200) antibody was from BD Pharmingen. For intracellular staining, the cells were stimulated with eBioscience cell stimulation cocktail (plus protein transport inhibitor) (eBioscience, 004975‐03) for 4 h, prior to staining antibodies against surface proteins, then fixed and permeabilized using a transcription factor staining buffer set (Thermo Fisher Scientific) and incubated with fluorescently labeled FITC‐conjugated antimouse TNF*α* (MP6‐XT22, 1:200), APC‐conjugated antihuman/mouse GranB (QA16A02, 1:200), PE‐conjugated antimouse IFN*γ* (XMG1.2, 1:200), FITC‐conjugated antihuman TNF*α* (MAb11, 1:200), and PE‐conjugated antihuman IFN*γ* (4S.B3, 1:200) antibodies. Multicolor FCM analysis was performed using a BD Fortessa 18 color analyzer. All data analysis was performed using the flow cytometry analysis program FlowJo v.10 (https://www.flowjo.com/).

### mRNA Library Construction

Total RNA was isolated using the TRIzol reagent. Oligo(dT)‐attached magnetic beads were used to purify mRNA. Purified mRNA was fragmented into small pieces with a fragment buffer at an appropriate temperature. First‐strand cDNA was generated using random hexamer‐primed reverse transcription, followed by second‐strand cDNA synthesis. Afterward, A‐Tailing Mix and RNA Index Adapters were added by incubating for end repair. The cDNA fragments obtained from the previous step were amplified by PCR, and products were purified using Ampure XP Beads, and then dissolved in EB solution. The product was validated using an Agilent Technologies 2100 bioanalyzer for quality control. The double‐stranded PCR products from the previous step were denatured and circularized using the splint oligo sequence to obtain the final library. Single‐strand circular DNA (ssCir DNA) was used as the final library. The final library was amplified with phi29 to form a DNA nanoball (DNB), which had more than 300 copies of one molecule, DNBs were loaded into the patterned nanoarray and single‐end 50 bases reads were generated on the BGIseq500 platform (BGI‐Shenzhen, China).

### RNA‐seq Analysis

The sequencing data were filtered with SOAPnuke (v1.5.2) by removing the 1) reads containing sequencing adapters, 2) reads whose low‐quality base ratio (base quality less than or equal to 5) was more than 20%, and 3) reads whose unknown base (“N” base) ratio was more than 5%. After this, the clean reads were obtained and stored in a FASTQ format. The clean reads were mapped to the reference genome using HISAT2 (v2.0.4). Ericscript (v0.5.5) and rMATS (V3.2.5) were used for fusion genes and differential splicing genes, respectively. Bowtie2 (v2.2.5) was used to align the clean reads to the gene set, a database for this organism was built by BGI (Beijing Genomic Institute in ShenZhen), coding transcripts were included, and the expression levels of genes were calculated using RSEM (v1.2.12). The heatmap was drawn using pheatmap (v1.0.8) according to the gene expression in different samples. Differential expression analysis was performed using DESeq2(v1.4.5) with a *Q* value ≤ 0.05. To gain insight into the change of phenotype, KEGG enrichment analysis of annotated differentially expressed genes was performed using Phyper based on the hypergeometric test. The heatmap and KEGG analyses results were plotted using the OmicShare tools, a free online platform for data analysis (www.omicshare.com/tools). The significance levels of terms and pathways were corrected by the *Q* value with a rigorous threshold (*Q* value ≤ 0.05) by Bonferroni correction.

### Statistical Analysis

All experiments were performed at least three times. Results are expressed as the mean ± SEM, and analyzed by two‐tailed unpaired Student's *t*‐test or one‐way analysis of variance (ANOVA), followed by LSD test. Flow cytometry data were analyzed by FlowJo v.10. Statistical analysis was conducted using GraphPad software (version 7.0). For survival analysis, Kaplan–Meier curves were used, and survival rates were determined using the log‐rank test. To analyze the correlation between the level of GPR54 and CD8^+^ T cell infiltration, the Tumor Immune Estimation Resource (TIMER2.0) web tool of the EPIC algorithm was applied. Statistical significance was set at *P* < 0.05, and denoted as *P < 0.05, **P < 0.01, and ***P < 0.001.

## Conflict of Interest

The authors declare no conflict of interest.

## Author Contributions

S.Z. and F.Y. contributed equally to this work. B.D., M.L., M.Q., Q. H., J.Q., and S.Z. designed the experiment. S.Z., F.Y., A.C., and Y.C. performed the experiment. S.Z., B.T., X.L., C.H., W.Z., C.M., and J.Q. analyzed the data. B.D., S.Z., and F.Y. wrote the manuscript. All authors contributed to discussing the results.

## Supporting information

Supporting Information

## Data Availability

The data that support the findings of this study are available on request from the corresponding author. The data are not publicly available due to privacy or ethical restrictions.
