# Supplementary material for: Neuroendocrine Regulation of Stress‐Induced T Cell Dysfunction during Lung Cancer Immunosurveillance via the Kisspeptin/GPR54 Signaling Pathway
Source: Adv Sci (Weinh). 2022 Feb 27;9(13):2104132. doi: 10.1002/advs.202104132 (PMC9069377; doi:10.1002/advs.202104132)
Supplement: Supplementary file 1 — Supporting Information [file ADVS-9-2104132-s001.pdf]

Supporting Information

Neuroendocrine regulation of stress-induced T cell dysfunction during lung cancer immunosurveillance via the kisspeptin/GPR54 signaling pathway

Su Zhang, Fangfei Yu, Anran Che, Binhe Tan, Chenshen Huang, Yuxue Chen, Qi Huang, Wenying Zhang, Chengbin Ma, Min Qian, Mingyao Liu, Juliang Qin*, Bing Du**

Figures

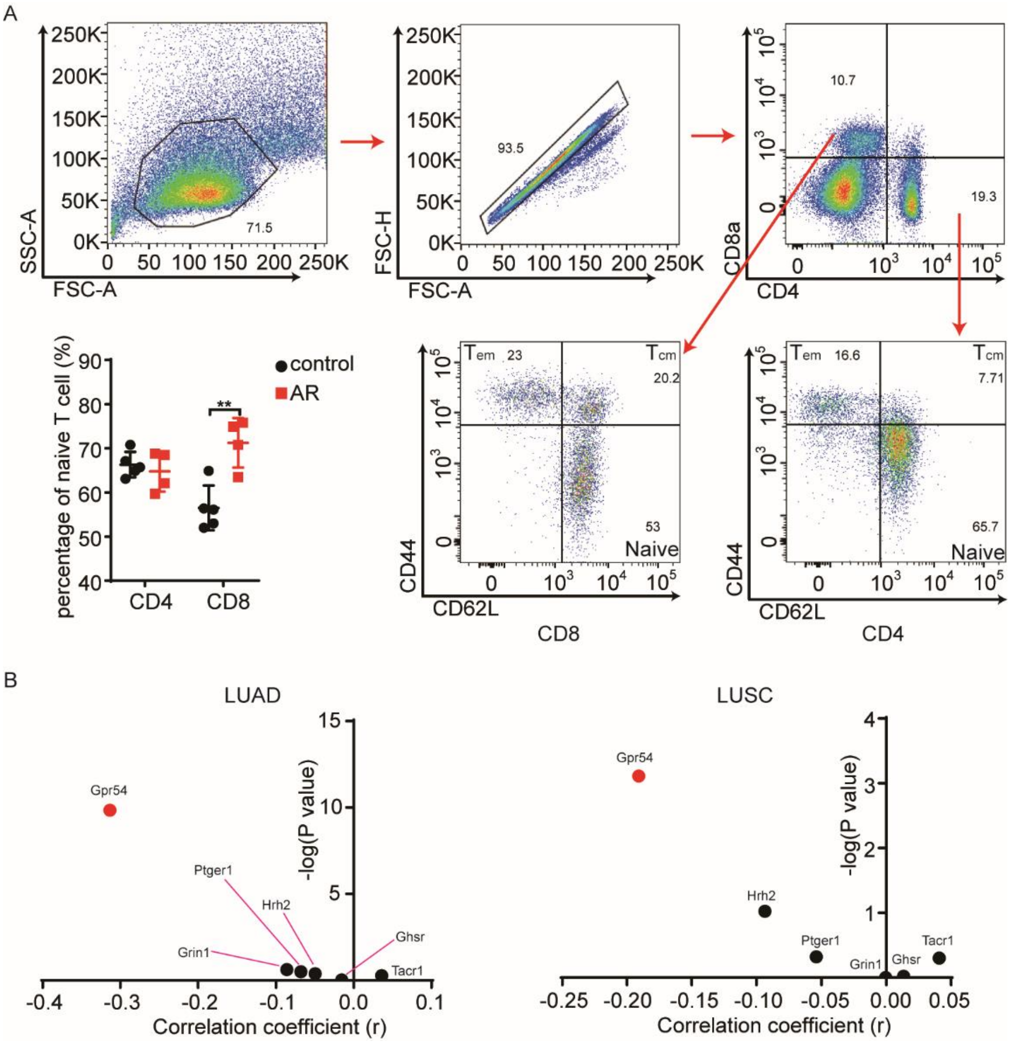

Figure S1. Representative gating strategies for splenic memory T cells. (A) Representative gating strategies for splenic central memory T cells (T_{cm}) and effector memory T cells (T_{em}) in Figure 1B-E, and the ratio of naïve T cells in splenic. (B)Scatter plots of gene expression in LUAD and LUSC of the TCGA database and $CD8^+$ T cell infiltration were generated using the Tumor Immune Estimation Resource (TIMER2.0) web tool of EPIC algorithm.

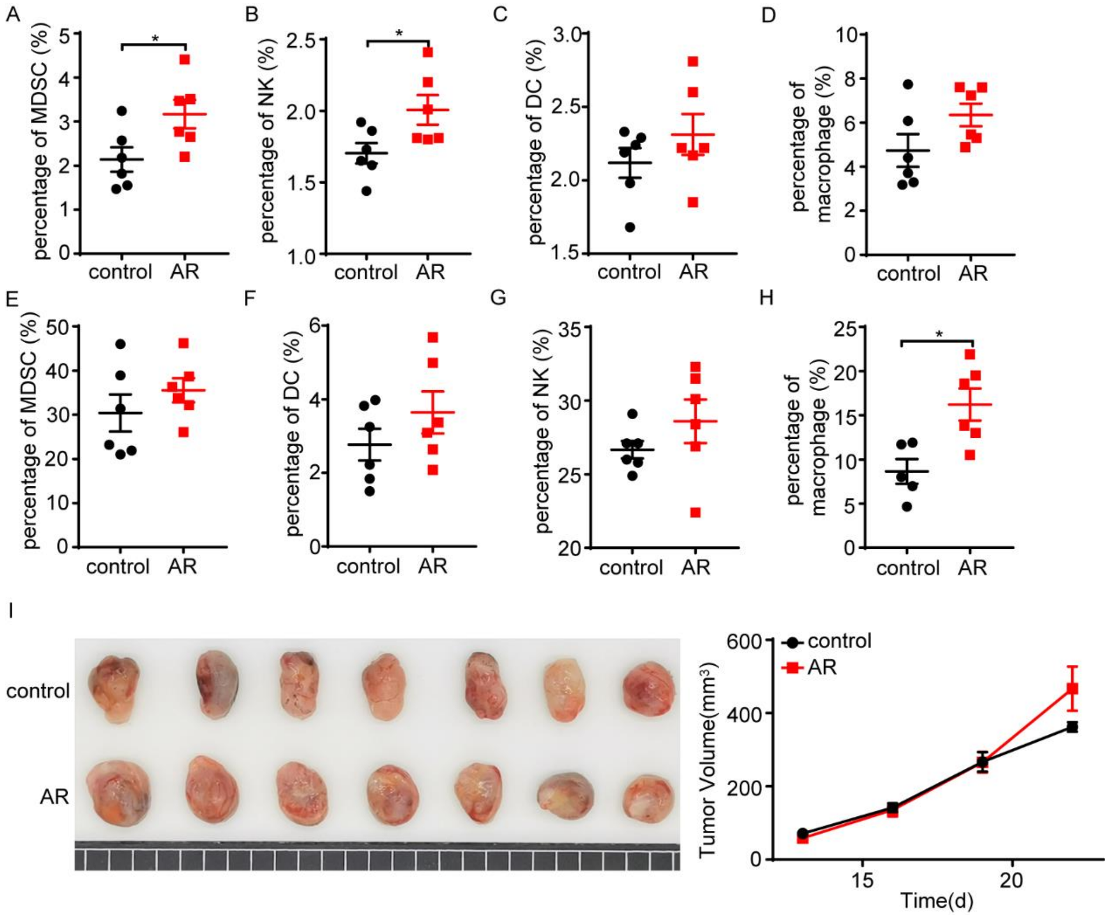

Figure S2. Chronic stress affects lung cancer through the adaptive immune system. FCM analysis of the proportions of spleen MDSC (A), NK (B), DC (C), macrophage (D) cell populations after 18 days of AR (n = 6); FCM analysis of the proportions of tumor MDSC (E), DC (F), NK (G), macrophage (H) cell populations at end-point (n = 6). $Rag2^{-/-}$ mice on day 18 after AR were then implanted with 10^6 LLC tumor cells by subcutaneous injection, and mice were subjected to AR treatment daily. The LLC tumor growth curves and the end-point tumor sizes (I) were represented (n = 7). All data are from at least three independent experiments. Data are represented as the mean ± SEM. *P < 0.05, **P < 0.01, ***P < 0.001 by an unpaired Student's *t*-test.

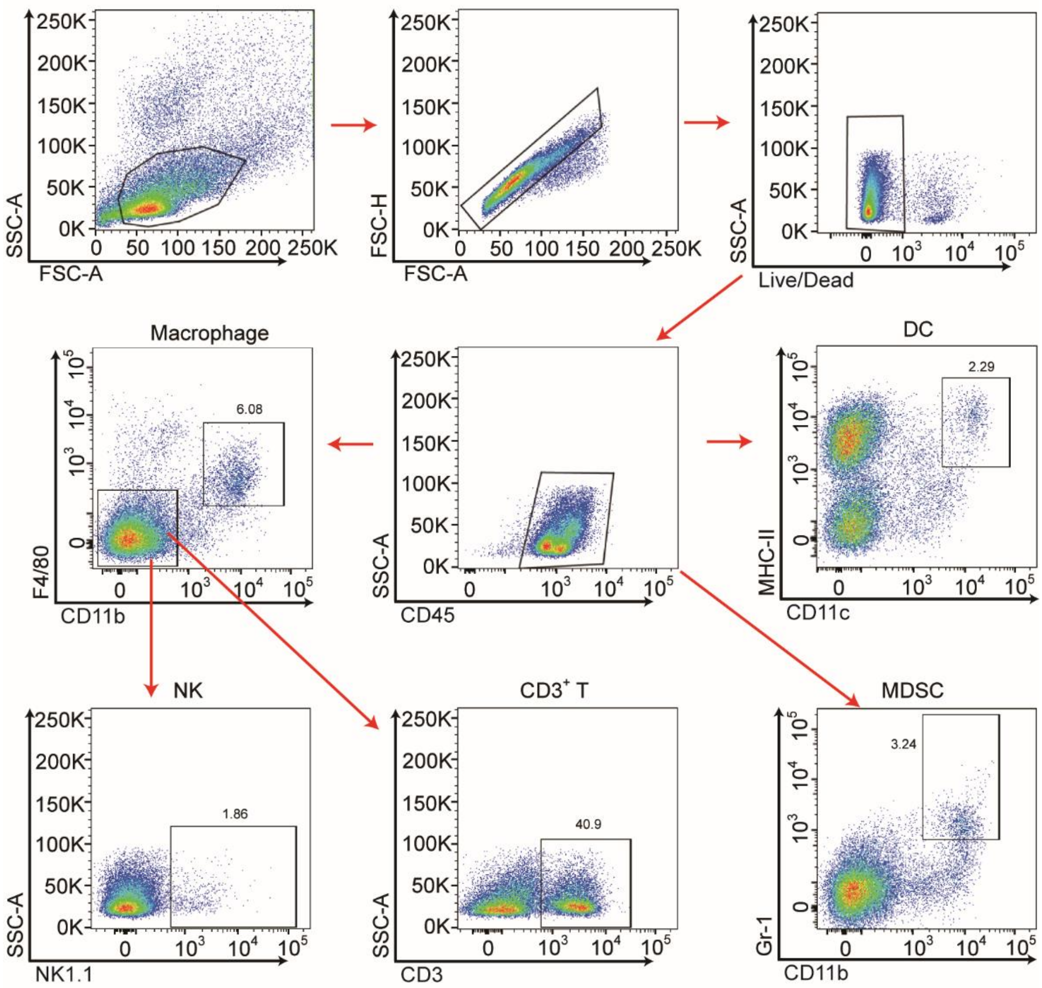

Figure S3. Representative gating strategies for all splenic immune cell panels.

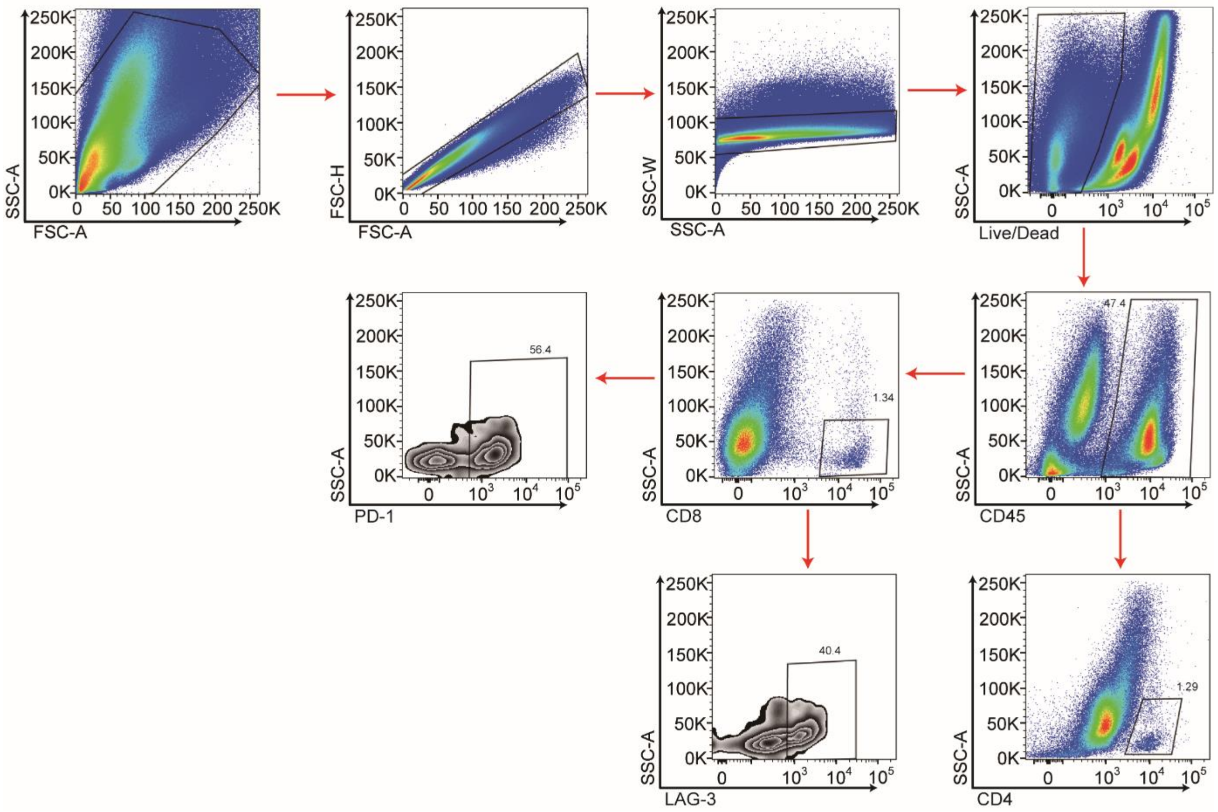

Figure S4. Representative gating strategies for all tumor T cells function panels.

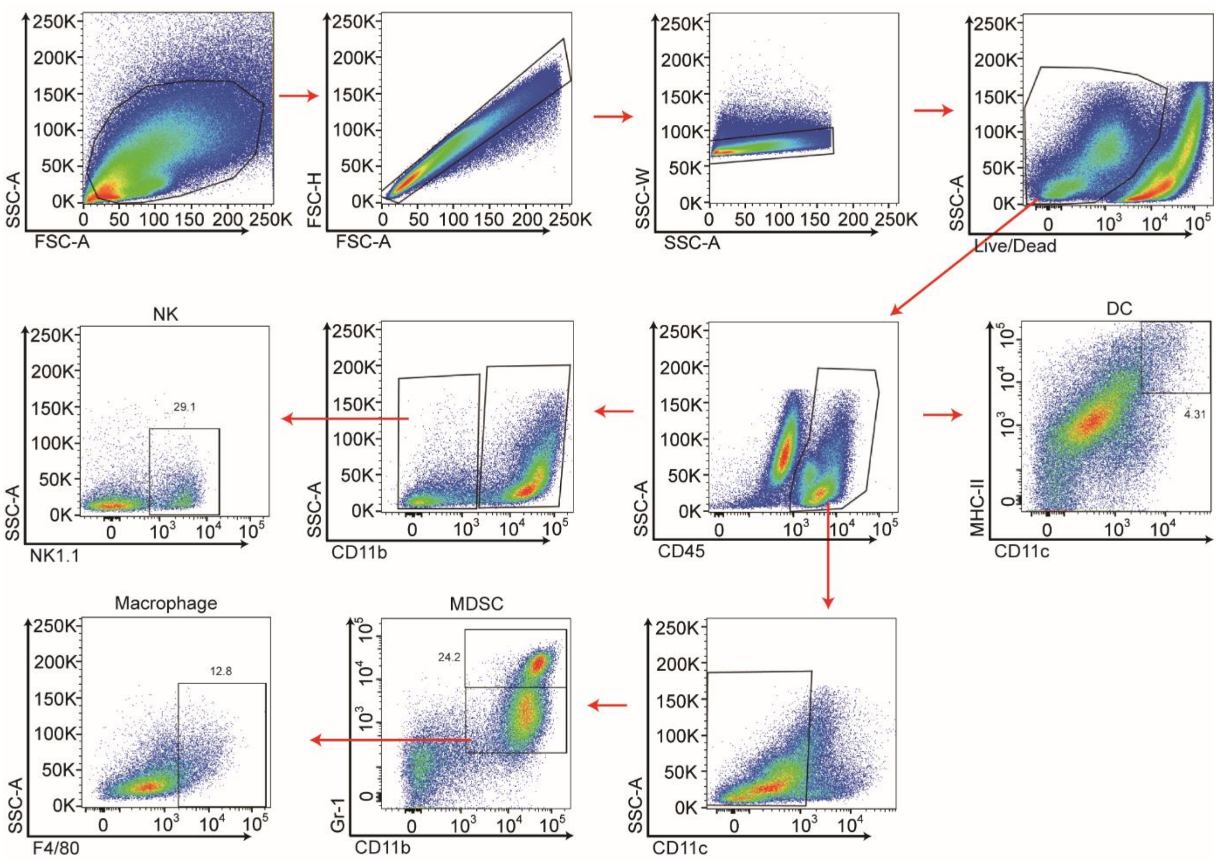

Figure S5. Representative gating strategies for all tumor immune cell panels.

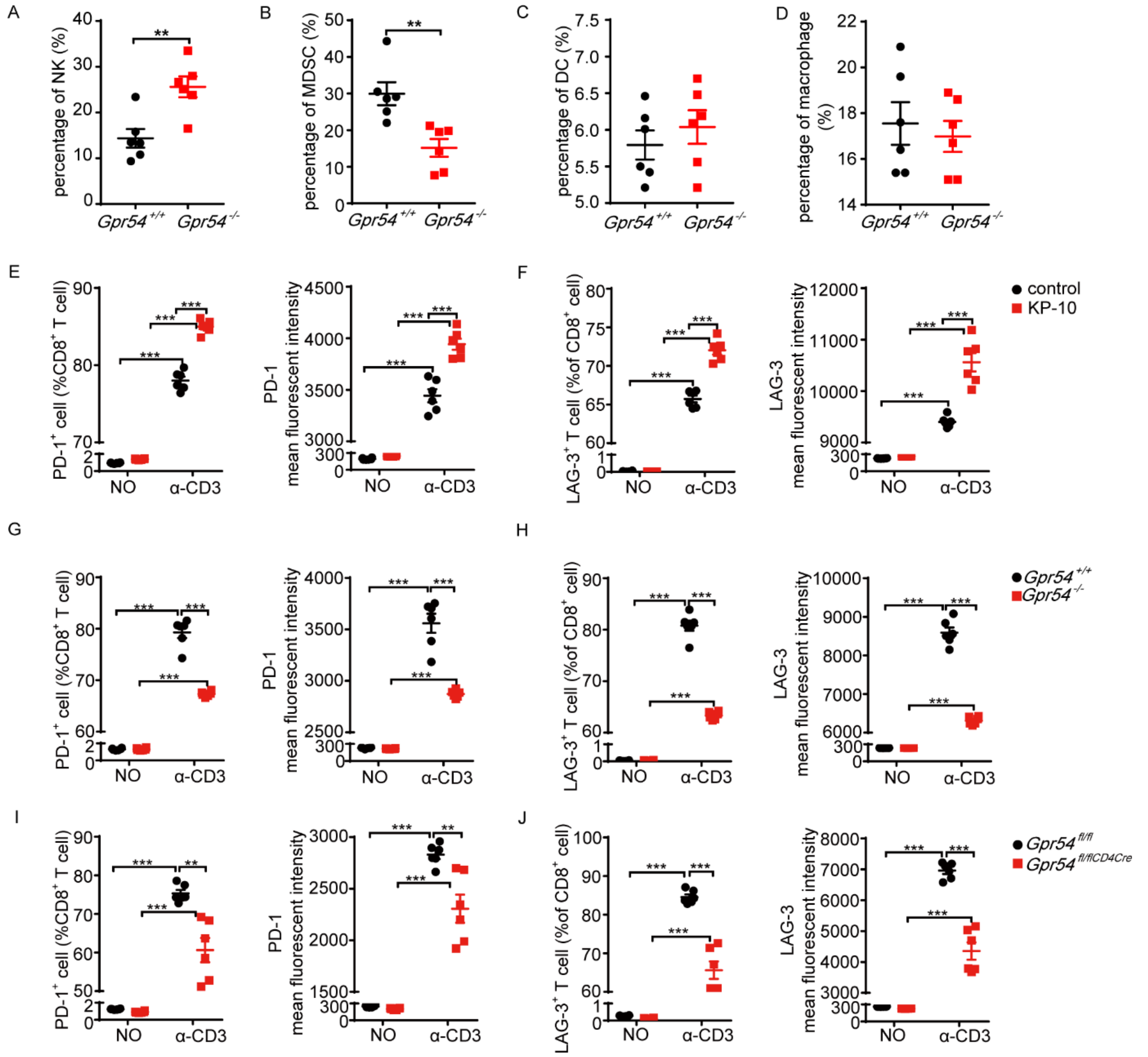

Figure S6. GPR54 promotes CD8^{+} T cell exhaustion. *Gpr54*$^{+/+}$ and *Gpr54*$^{-/-}$ mice implanted with 10^{6} LLC tumor cells by subcutaneous injection, FCM analysis of the proportions of tumor NK (A), MDSC (B), DC (C), and macrophage (D) cell populations at end-point (n = 6); Expression of PD-1 (E) and LAG-3 (F) of mice CD8^{+} T cell after pretreat with KP-10 (10 μM) 6 h then re-stimulation with α-CD3 (1 μg/ml) for 72 hours (n = 6); Expression of PD-1 (G) and LAG-3 (H) of *Gpr54*$^{+/+}$ and *Gpr54*$^{-/-}$ mice CD8^{+} T cell after re-stimulation with α-CD3 (1 μg/ml) for 72 hours (n = 6); Expression of PD-1 (I) and LAG-3 (J) of *Gpr54*$^{fl/fl}$ and *Gpr54*$^{fl/flCD4Cre}$ mice CD8^{+} T cell after re-stimulation with α-CD3 (1 μg/ml) for 72 h (n = 6). All data are from at least three independent experiments. Data are represented as the mean ± SEM. *P < 0.05, **P < 0.01, ***P < 0.001 by an unpaired Student's *t*-test.

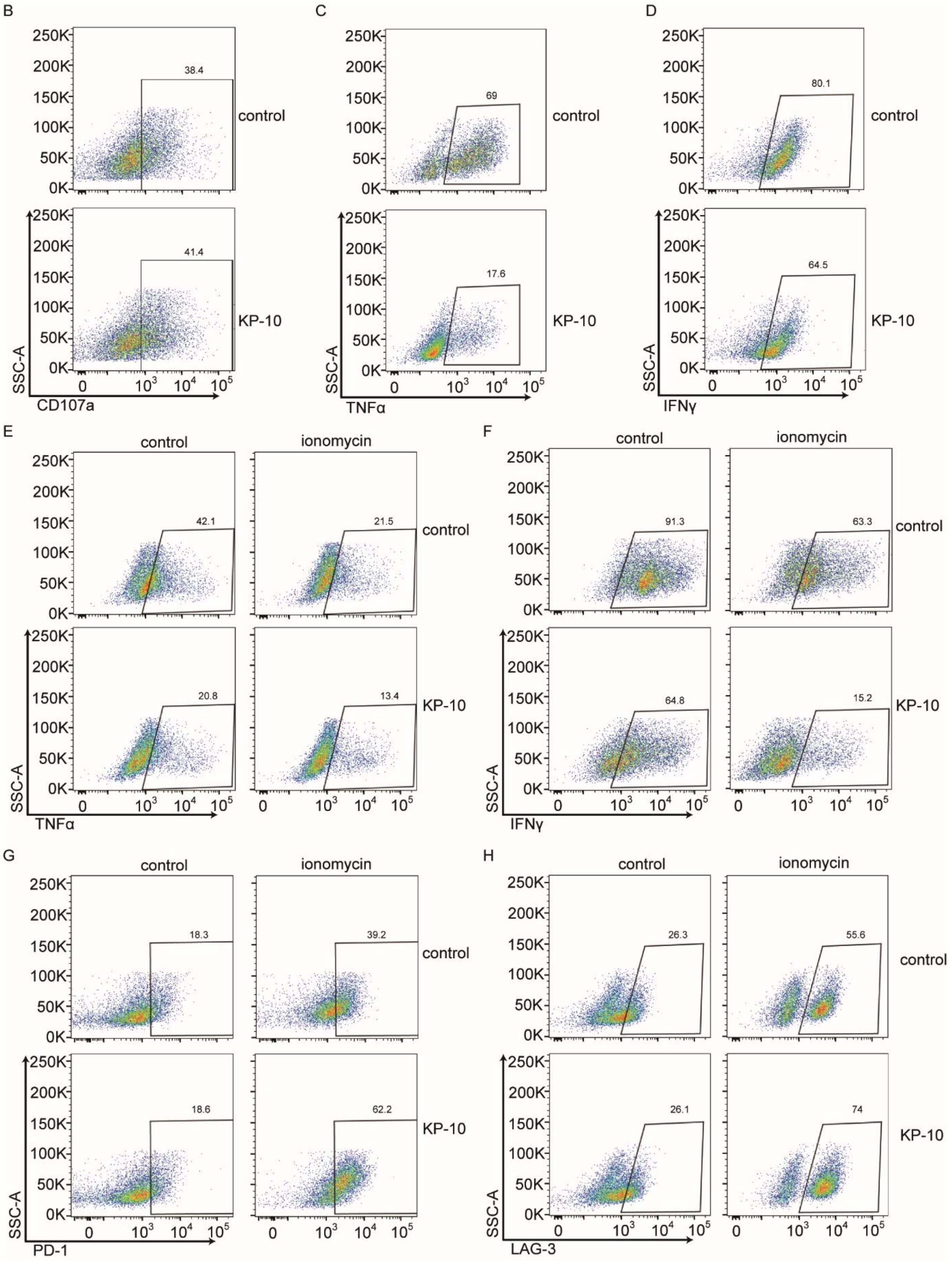

Figure S7. Representative plot for Figure 3B-H.

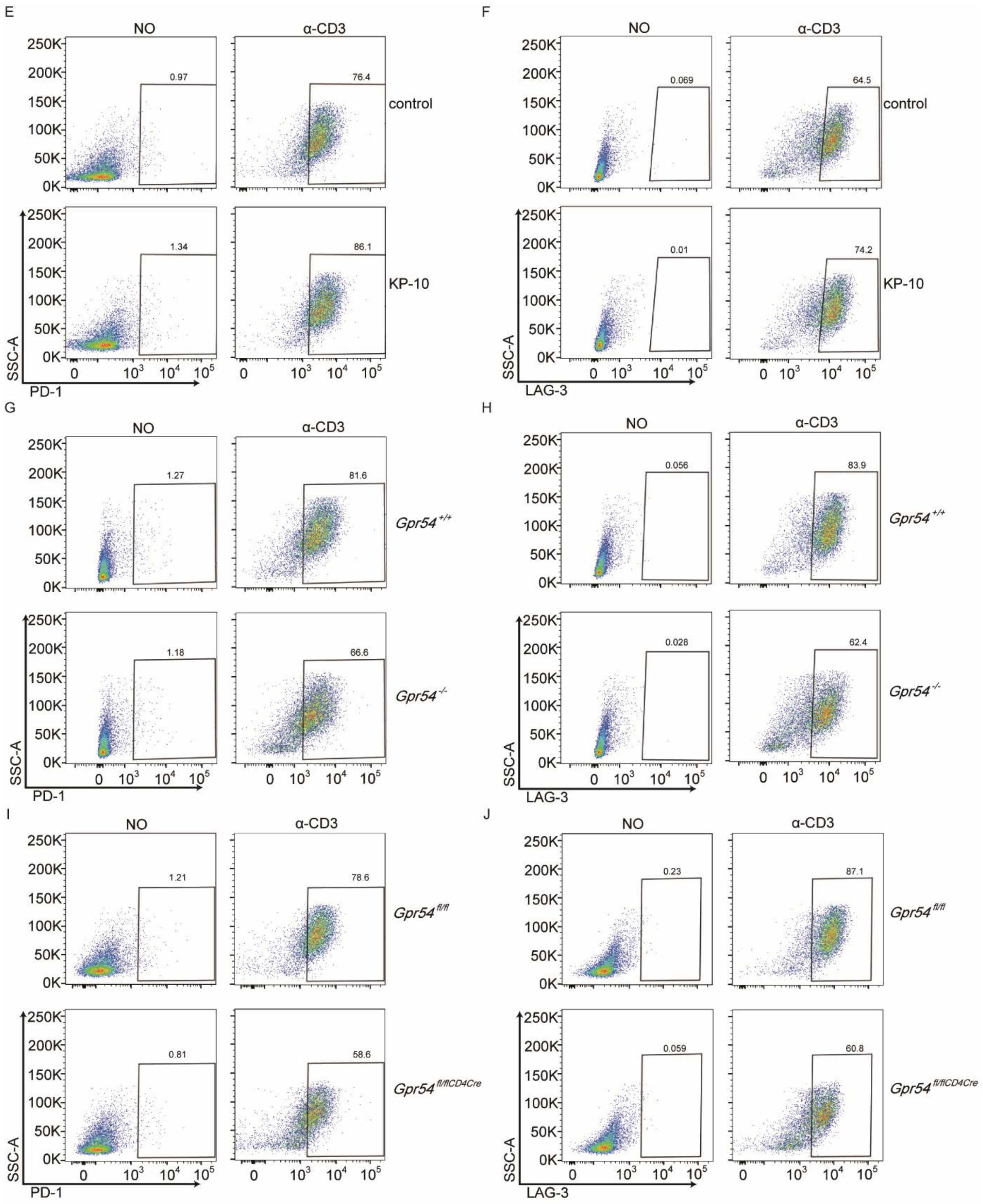

Figure S8. Representative plot for Figure S2E-J.

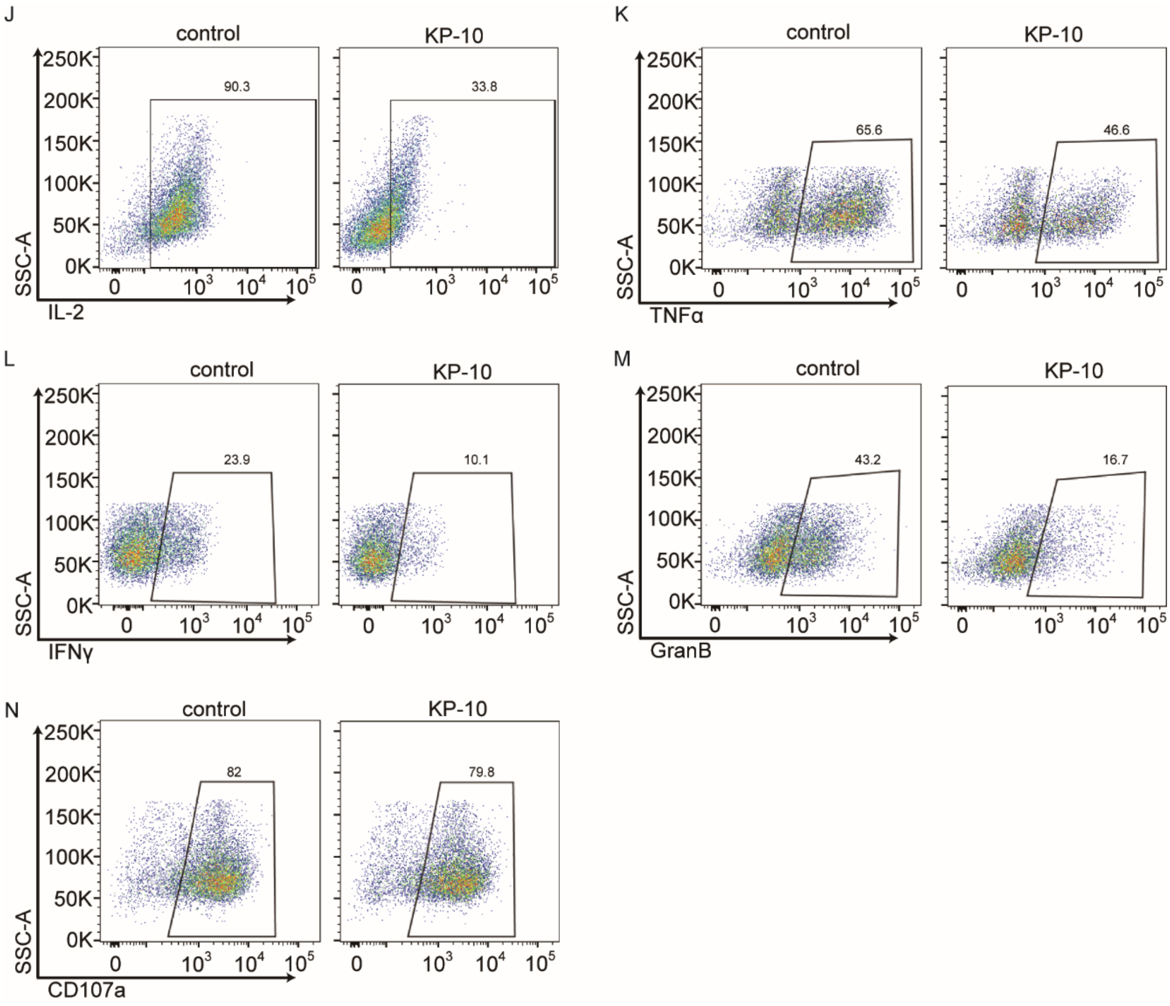

Figure S9. Representative plot for Figure 3J-N.

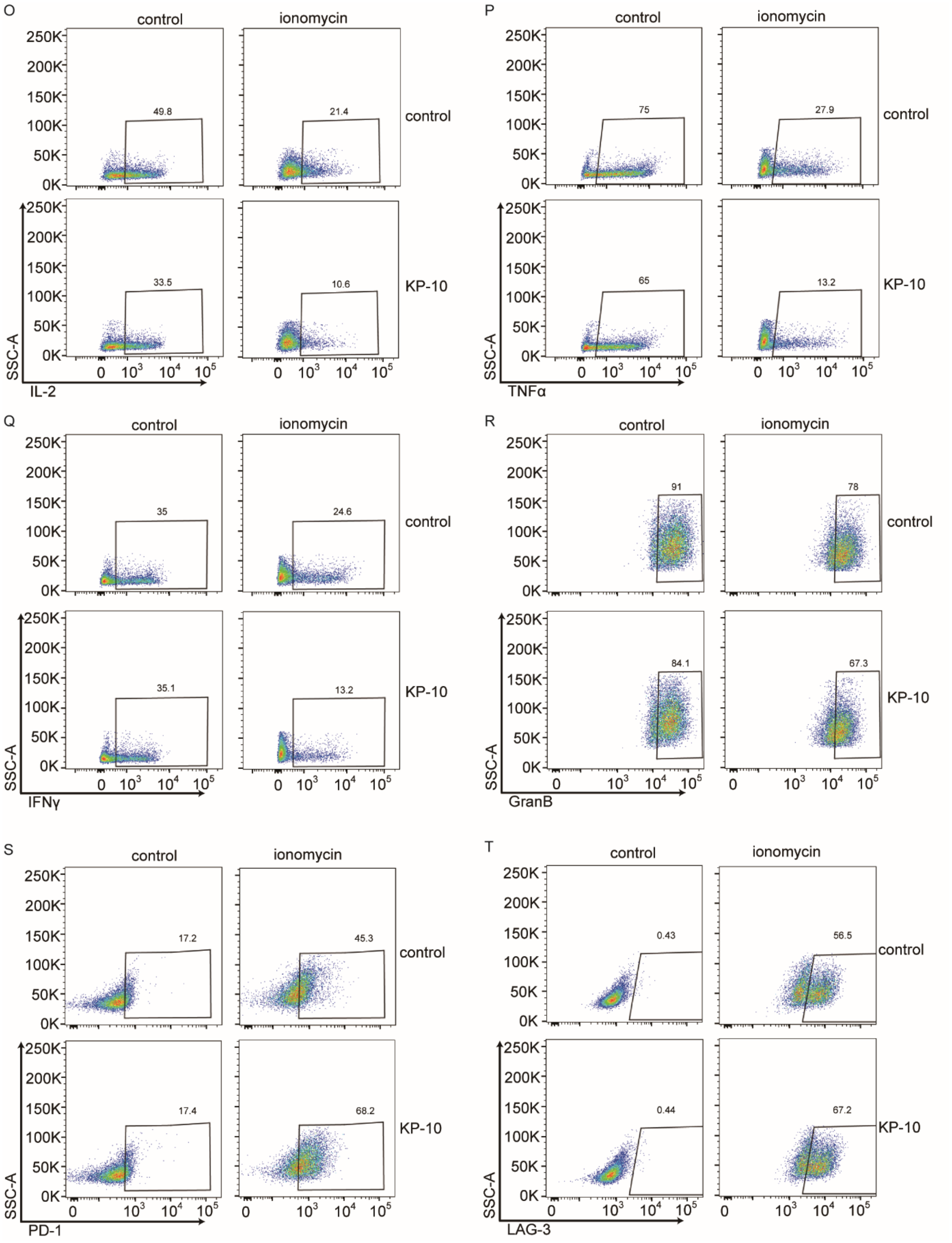

Figure S10. Representative plot for Figure 3O-T.

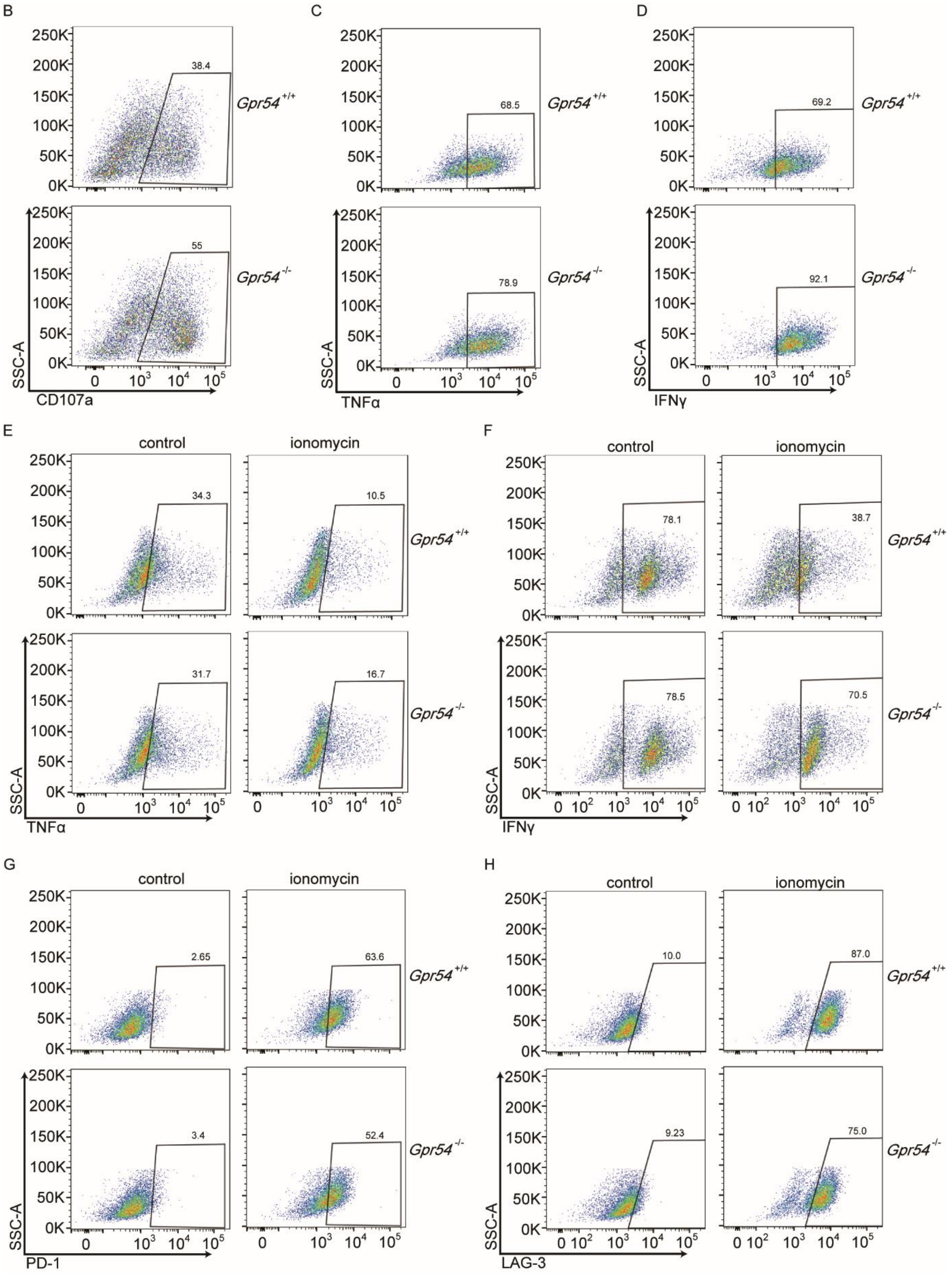

Figure S11. Representative plot for Figure 4B-H.

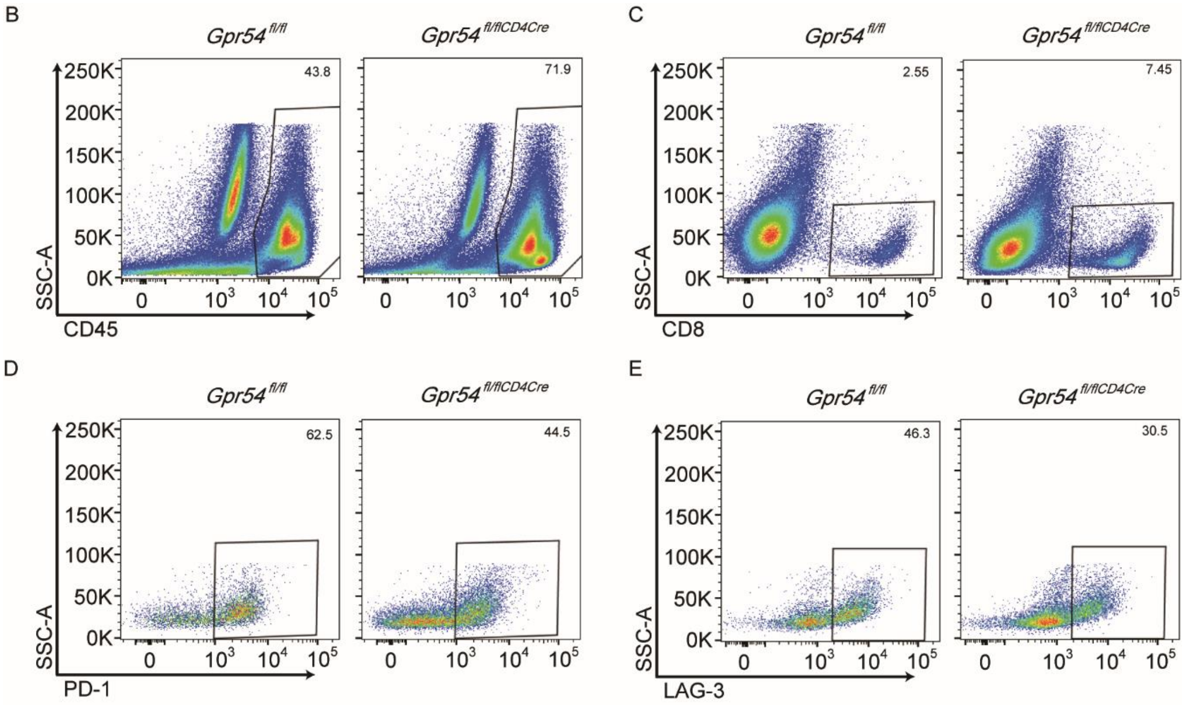

Figure S12. Representative plot for Figure 5B-E.

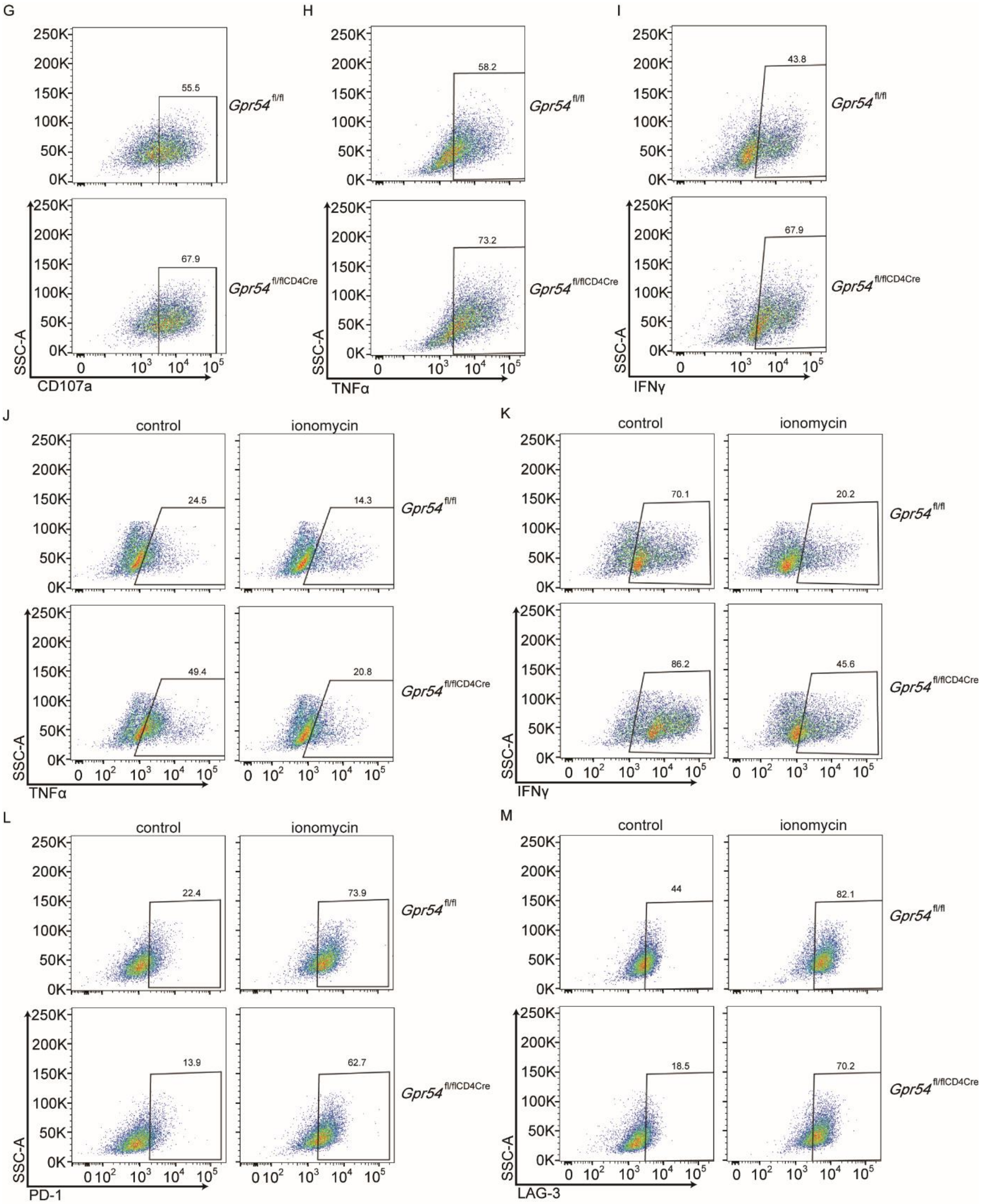

Figure S13. Representative plot for Figure 5G-M.

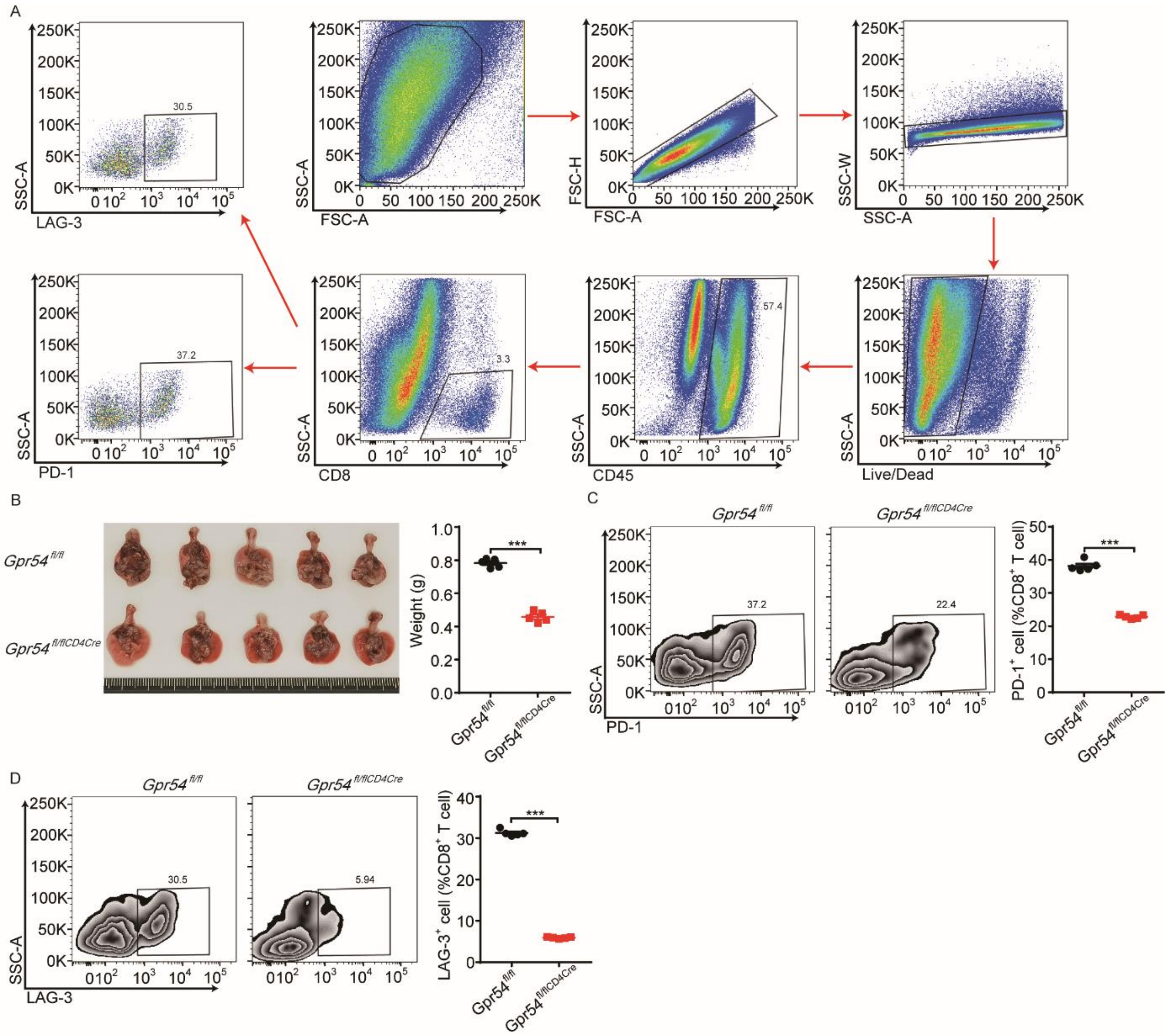

Figure S14. GPR54 T cell conditional knockout impairs orthotopic lung tumor development and decreases CD8$^+$ T cell exhaustion. *Gpr54*$^{fl/fl}$ and *Gpr54*$^{fl/flCD4Cre}$ mice were implanted with 2×10^5 LLC tumor cells into the left lung lobe by intercostal injection at the median axillary line. (A) Representative gating strategies for lung T cell function panels. (B) The lung picture and the weight of lung at end-point were represented. FCM analysis of the proportions of lung PD-1$^+$ CD8$^+$ (C), and LAG-3$^+$ CD8$^+$ (D) cell populations at end-point (n = 5). All data are from at least three independent experiments. Data are represented as the mean ± SEM. ***P < 0.001 by an unpaired Student's *t*-test.

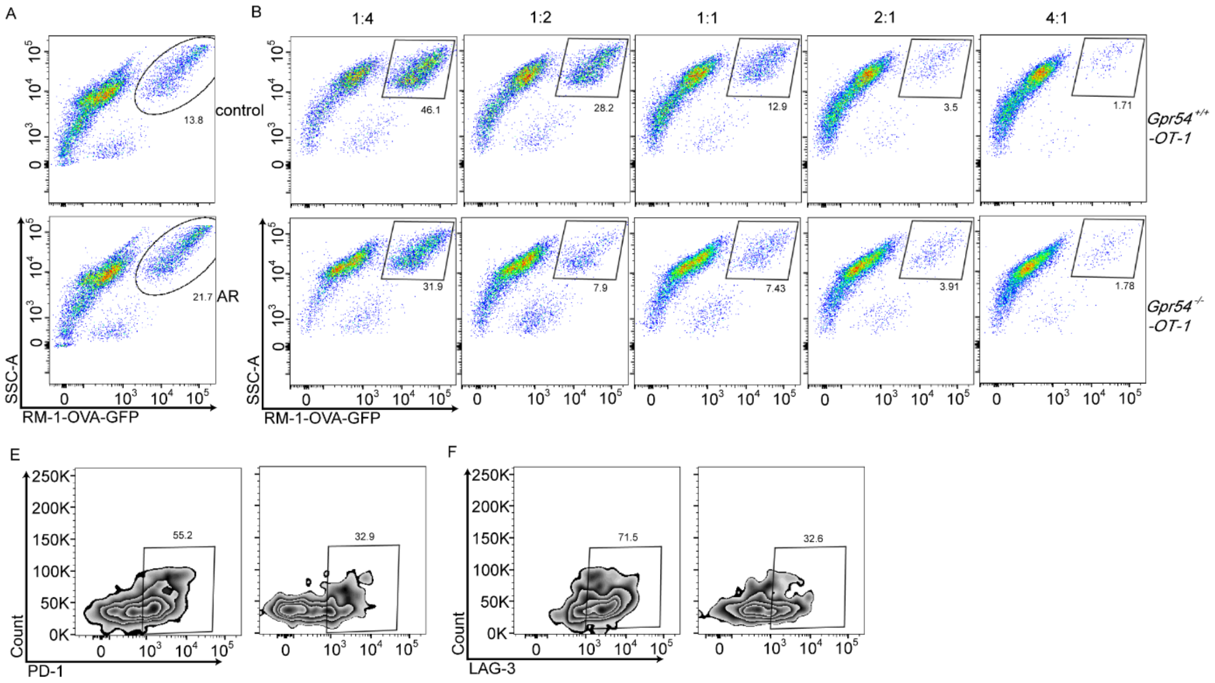

Figure S15. Representative plot for Figure 6A, B, E and F.

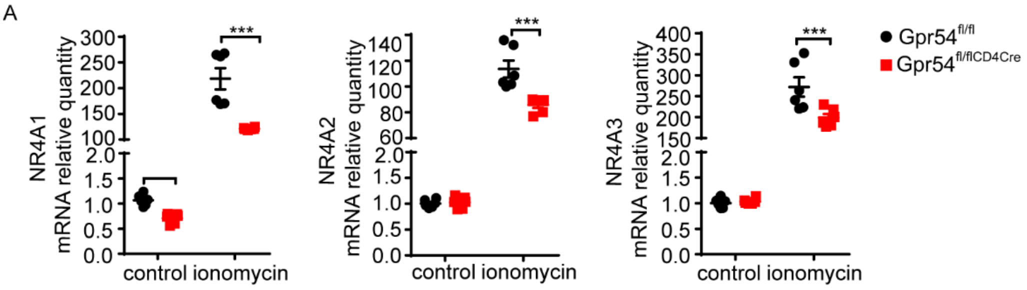

Figure S16. GPR54 promotes NR4A expression. *Gpr54*$^{fl/fl}$ and *Gpr54*$^{fl/flCD4Cre}$ mouse splenic CD8^{+} T cells activated with α-CD3 (5 μg)/α-CD28 (2 μg) for 72 h, then treated with ionomycin for 16 h, Gene expression of splenic CD8^{+} T with ionomycin was determined by qPCR (n = 6). All data are from at least three independent experiments. Data are represented as the mean ± SEM. *P < 0.05, **P < 0.01, ***P < 0.001 by one-way ANOVA followed by LSD analysis.

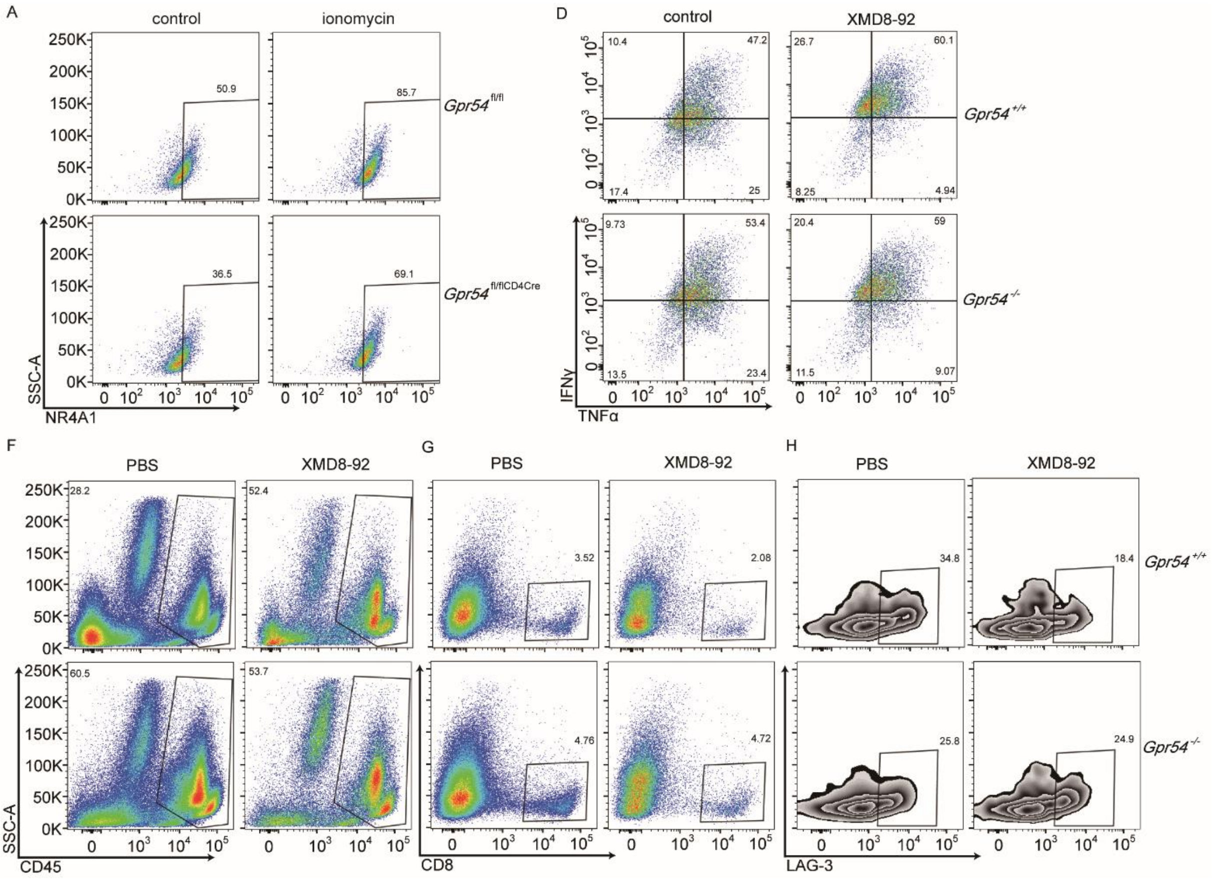

Figure S17. Representative plot for Figure 7A, D, and F-H.

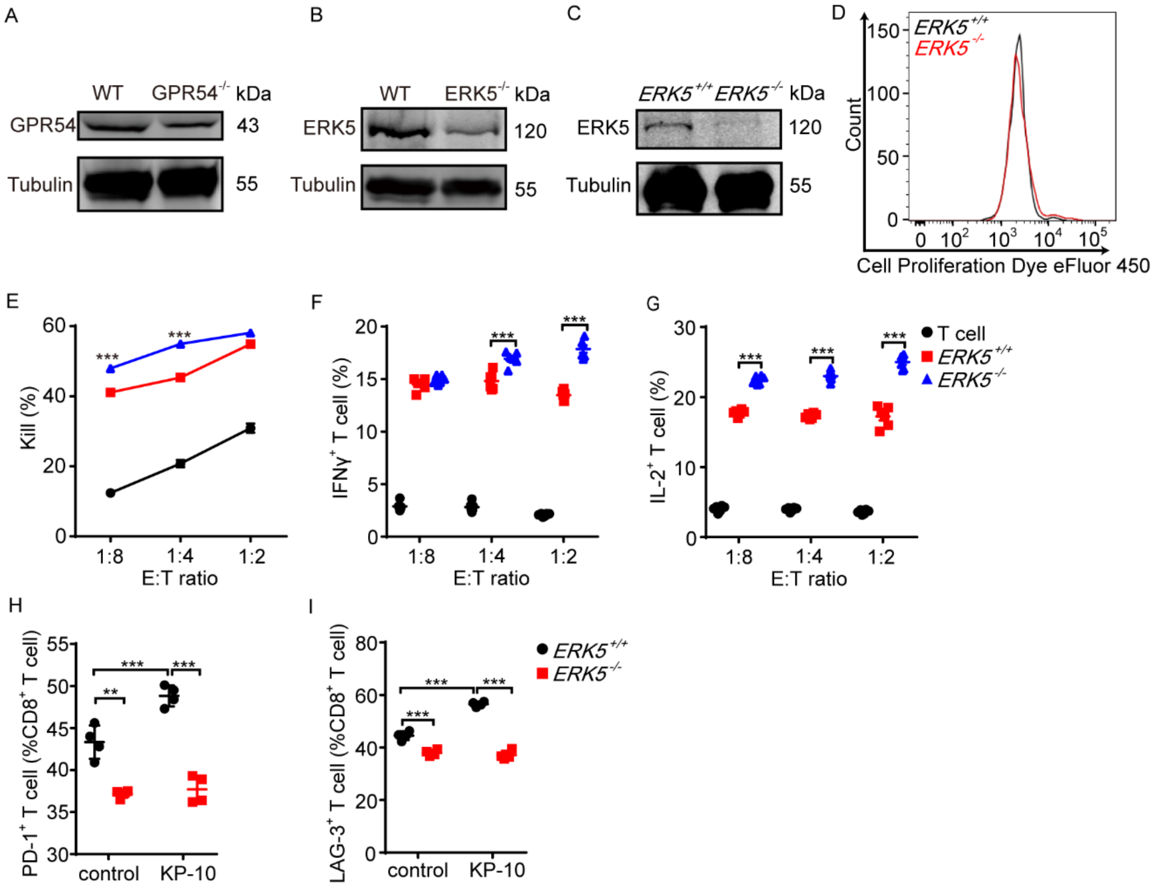

Figure S18. *ERK5* deletion enhance CD19 CAR-T cell function. GPR54 (A) and ERK5 (B) expression after CRISPR/Cas9 engineering in PSMA-CAR-T were determined by Western blot. (C) ERK5 expression in CD19-CAR-T was analyzed by immunoblotting. (D) CD19 CAR-T cells were stained with 5 μM cell proliferation dye eFluor 450, and 72 h later cell proliferation was detected by flow cytometry. Raji cells with or without KP-10 (10 μM) were mixed with T, *ERK5*$^{+/+}$ or *ERK5*$^{-/-}$ CD19 CAR-T cells at the indicated effector to target (E:T) ratios, cytotoxicity assay (E), cytokine release (F, G), and exhaustion (H, I) were presented (n = 6). All data are from at least three independent experiments. Data are represented as the mean ± SEM. **P<0.01, ***P < 0.001 by an unpaired Student's *t*-test (F, G) or one-way ANOVA followed by LSD analysis (H, I).

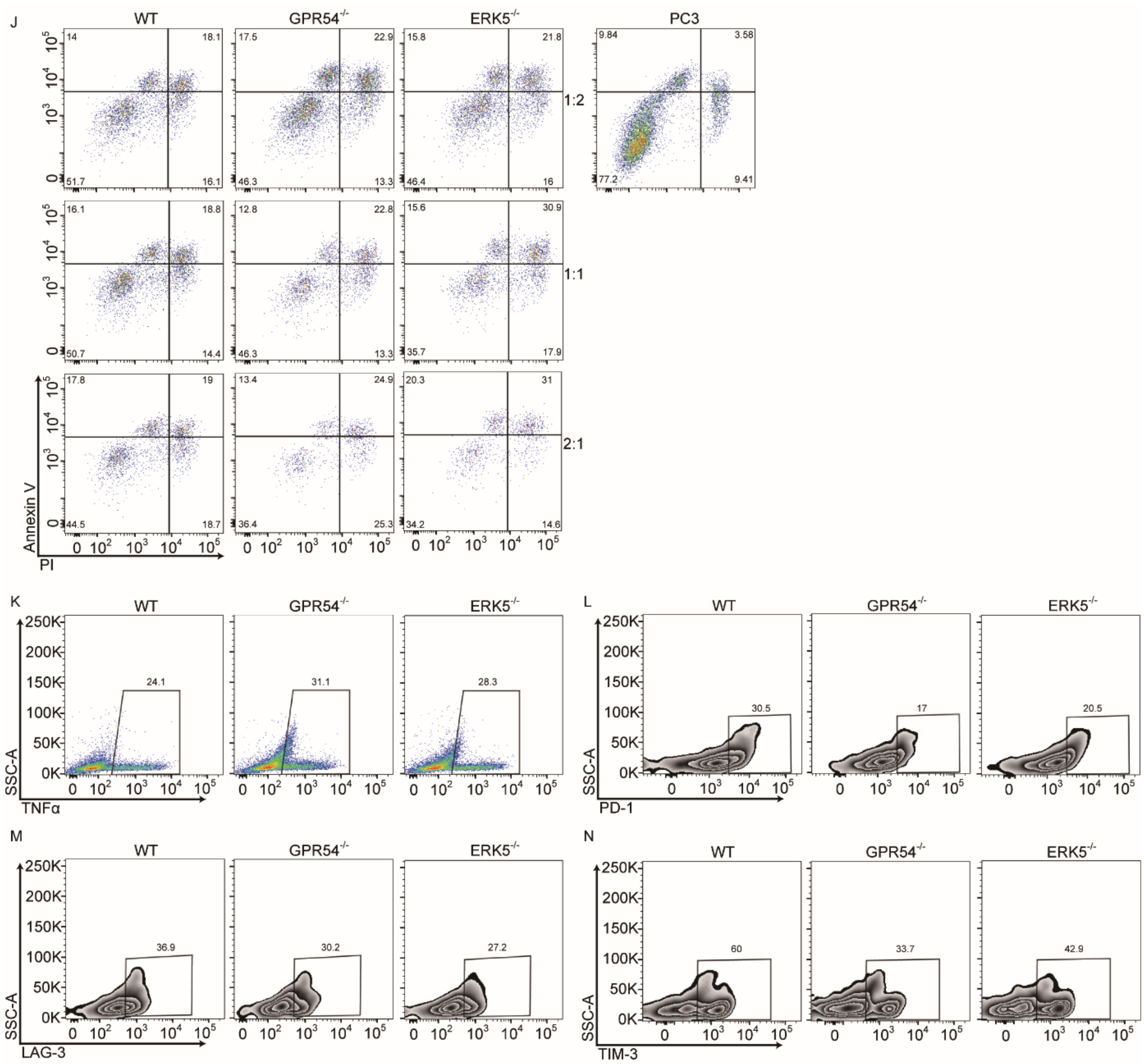

Figure S19. Representative plot for Figure 7J-N.

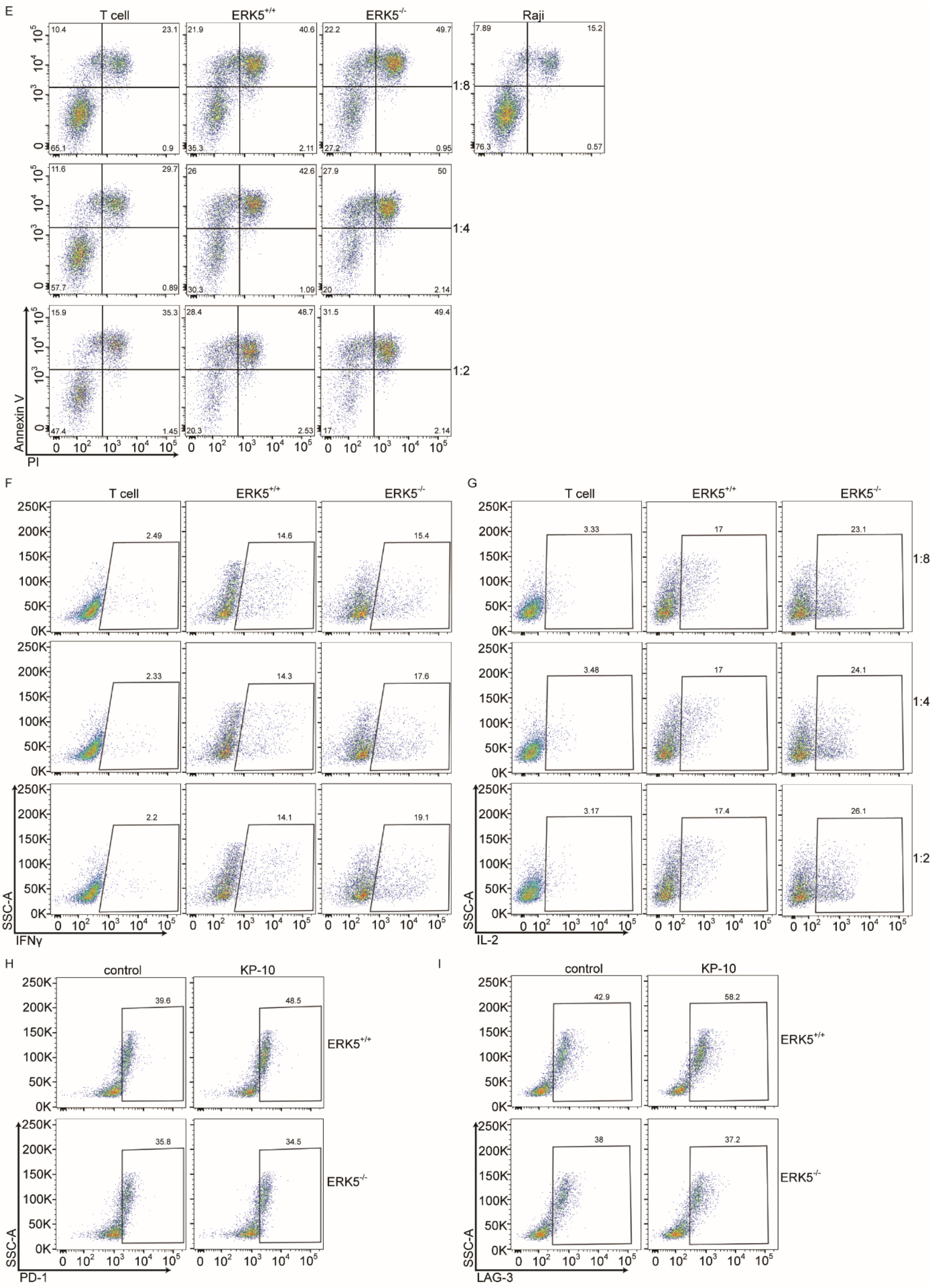

Figure S20. Representative plot for Figure S5E-I.
